# Identification of indications for albumin administration in septic patients with liver cirrhosis

**DOI:** 10.1186/s13054-023-04587-3

**Published:** 2023-07-28

**Authors:** Wenhan Hu, Hui Chen, Chencheng Ma, Qin Sun, Meicheng Yang, Haofei Wang, Qingyun Peng, Jinlong Wang, Chen Zhang, Wei Huang, Jianfeng Xie, Yingzi Huang

**Affiliations:** 1 Jiangsu Provincial Key Laboratory of Critical Care Medicine, Department of Critical Care Medicine, Zhongda Hospital, School of Medicine, Southeast University, No. 87, Dingjiaqiao Road, Gulou District, Nanjing, 210009 People’s Republic of China; 2 Department of Critical Care Medicine, The First Affiliated Hospital of Soochow University, Soochow University, No. 899 Pinghai Road, Suzhou, 215000 People’s Republic of China; 3 The State Key Laboratory of Bioelectronics, School of Instrument Science and Engineering, Southeast University, Nanjing, People’s Republic of China

**Keywords:** Sepsis, Liver cirrhosis, Albumin, Time-varying confounder

## Abstract

**Background:**

Albumin infusion is the primary therapeutic strategy for septic patients with liver cirrhosis. Although recent studies have investigated the efficacy of albumin in the resuscitation stage of septic patients with liver cirrhosis, it remains unclear whether daily albumin administration can improve outcomes. Furthermore, the indications for initiating albumin therapy are not well defined.

**Methods:**

Septic patients with liver cirrhosis were obtained from the Medical Information Mart for Intensive Care (MIMIC-IV 2.0) database. Marginal structural Cox models were employed to investigate the association between daily albumin infusion and 28-day mortality. We also aimed to explore under what circumstances enrolled patients could benefit most from albumin administration, based on the clinical parameters collected on the day of albumin infusion, including serum albumin concentration, serum lactate concentration, mean arterial pressure (MAP), and vasopressor dosage.

**Results:**

A total of 2265 patients were included in the final analysis, of whom 1093 (48.3%) had received albumin treatment at least once. The overall 28-day mortality was 29.6%. After marginal structural modeling, daily albumin infusion was associated with a reduced risk of 28-day death (hazard ratio, 0.76; 95% CI 0.61–0.94). We found that patients benefit most from albumin infusion when initiated on the day of serum albumin concentration between 2.5 and 3.0 g/dL, serum lactate concentration greater than or equal to 2 mmol/L, MAP less than 60 mmHg, or vasopressor dosage between 0.2 and 0.3 mcg/kg/min (norepinephrine equivalent, NEE).

**Conclusions:**

Albumin infusion is associated with a reduction in mortality in septic patients with liver cirrhosis under specific circumstances. Serum albumin concentration, serum lactate, MAP, and vasopressor dosage were found to be modifiers of treatment effectiveness and should be considered when deciding to initial albumin infusion.

**Supplementary Information:**

The online version contains supplementary material available at 10.1186/s13054-023-04587-3.

## Background

Sepsis is defined as the dysregulated host response to infection that leads to life-threatening acute organ dysfunction [1, 2]. Liver cirrhotic patients with sepsis have worse outcomes than those without, mainly attributed to its intrinsic characteristics, including portosystemic shunting, gut dysbiosis, increased bacterial translocation, cirrhosis-associated immune dysfunction, and genetic factors [3, 4]. Cirrhotic patients with sepsis constitute a distinct population regarding clinical course and prognosis, mainly characterized by prominent hemodynamic instability, reduced colloid oncotic pressure, and hypoalbuminemia. Thus, albumin infusion is pivotal to improving this population's prognosis, which they are prone to benefit from [5, 6].

Although albumin administration demonstrated its potential beneficial effects for cirrhotic patients with sepsis in several clinical trials [7, 8], and its role in reversing sepsis-induced hypotension is endorsed by current guidelines [9], several controversial and challenging issues remain to be addressed. First, recent studies investigating the efficacy of albumin in septic patients with liver cirrhosis tend to focus on the resuscitation stage, neglecting the impact on the prognosis of dynamic albumin administration throughout the entire clinical course of the illness. Aside from maintaining intravascular oncotic pressure during resuscitation, albumin’s anti-inflammatory, immunomodulating, positive effects on vessel wall integrity, drug-carrying, and nitric oxide scavenging properties can also lead to more favorable outcomes. Second, although limited data pointed out a potential benefit of maintaining serum albumin concentration at more than 30 g per liter in critically ill patients [10, 11], the well-designed ALBIOS trial could not substantiate the advantages of albumin infusion, a conclusion that may partly be attributed to excluding patients with cirrhosis [12]. For septic patients with liver cirrhosis, a specific cutoff for serum albumin concentration has not been established yet, below which initiating albumin administration would be invariably associated with lower mortality and considered a therapeutic necessity. Moreover, whether cutoff values derived from clinical variables other than serum albumin concentration can be used as indications for albumin administration has not been explored thoroughly.

This study focused primarily on patients with sepsis and liver cirrhosis, aiming to investigate dynamic albumin administration's impact on prognosis and clarify potential indications for albumin administration.

## Materials and methods

### Study design and participants

We conducted a retrospective analysis of the MIMIC-IV (2.0) database, which contains comprehensive and high-granularity information about well-defined and characterized patients admitted to ICUs at Beth Israel Deaconess Medical Center between 2008 and 2019 [13]. Two authors obtained access to the database and were responsible for data extraction (certification numbers 27252652 and 39732765).

Critically ill adult patients with liver cirrhosis who met the Sepsis 3.0 criteria were eligible (Supplemental Method A) [2, 14, 15]. Patients with a hospital length of stay less than 24 h, those who were identified as sepsis 12 h before or 24 h after ICU admission, and patients with an ICU length of stay less than 24 h or more than 100 days were excluded. Furthermore, we analyzed only the first ICU stay for patients who were admitted to the ICU more than once.

Because only third-party anonymized publicly available data were used, the study was considered exempt from human subjects committee review. This study was reported in accordance with the REporting of studies Conducted using Observational Routinely-collected health Data (RECORD) Statement [16].

### Variable extraction and data collection

Three sets of data were collected: baseline, daily observations, and outcome. The following data were extracted from the MIMIC-IV database on the first day of ICU admission: age, gender, weight, ethnicity, admission type, comorbidity, Sequential Organ Failure Assessment (SOFA) score, and each component of the SOFA score. Other relevant data, including vital signs, laboratory measurements, and treatment regimens, were obtained daily throughout the ICU stay. If a variable was recorded more than once on one ICU Day, we used the value related to the greatest severity of illness. The selection strategy for variables with multiple measurements is shown in Additional file 1: Table S1. The chart time of measurement and physiologic values were extracted from the database. Based on Townsend et al. [17], we conducted a thorough identification of infection sites based on the ICD-9-CM and ICD-10-CM codes available at discharge (Additional file 1: Table S2). Septic shock was recognized as vasopressor use and a serum lactate concentration > 2 mmol/L. Acute kidney injury was defined according to the clinical practice guidelines of Kidney Disease: Improving Global Outcomes (KDIGO) [18]. Time to antibiotics was determined as the duration from ICU admission to antibiotic administration. Variables with more than 60% missing values were excluded from the analysis (Additional file 1: Table S3, Table S4) [19]. Multiple imputation by weighted predictive mean matching was performed for variables with missing values of less than 60% (Supplemental Method B) [20].

### Primary exposure and outcomes

The main exposure of interest was the daily administration of intravenous (IV) albumin throughout the ICU stay. All manners of administering albumin were considered. The infusion time of albumin, solution concentration, and the total amount of albumin administered were extracted from the database.

The primary outcome was 28-day mortality. Secondary outcomes included ICU-free days at day 28, hospital-free days at day 28, and in-hospital mortality, defined as the status of patient survival at the time of hospital discharge.

### Statistical analysis

The study population was categorized into those treated with albumin (albumin group) and those who did not receive albumin during the entire ICU stay (non-albumin group). Values are presented as the mean (standard deviation) or median [interquartile range (IQR)] for continuous variables when appropriate and as the total number (percentage) for categorical variables. Comparisons between groups were made using the X^2^ test or Fisher’s exact test for categorical variables and Student’s t-test or Mann–Whitney U test for continuous variables as appropriate.

Albumin exposure was initially dichotomized as “any dosage versus none” on a daily basis. To estimate the impact of albumin treatment on 28-day mortality from longitudinal observational data, the time-dependent nature of albumin administration was first explored by marginal structural Cox proportional hazards models (MSCM) with inverse probability weighting (Supplemental Method C) [21]. The probability of receiving albumin infusion was weighted by adjusting for baseline confounders (age, gender, admission type, ethnicity, infection site, and serum albumin concentration) and time-varying confounders (SOFA score, serum lactate concentration, PaO_2_/FiO_2_ ratio, vasopressor dose, daily UO, and MAP) (Additional file 1: Figure S1). An extended Kaplan–Meier survival analysis was also performed, incorporating the weights derived from the marginal structural models [22]. As the effect of albumin treatment may differ depending on the time of initiation, MSCM with Heaviside functions was utilized to assess how the hazard ratio (HR) for death at 28 days changed over four consecutive weeks [23]. Each follow-up interval was assigned its own HR. Further, the correlation was re-evaluated in a multivariable Cox model with time-fixed and time-dependent covariates for 28-day mortality adding to the model the effect of albumin administration, weighted with marginal structural models [24]. In multivariable MSCM, we avoided multicollinearity by removing highly correlated covariates (Additional file 1: Figure S2, Figure S3) and identified the subset of covariates that gave the lowest -2 log L value by stepwise selection (Additional file 1: Figure S4). At last, extended Cox proportional hazards models with time-varying confounders were adopted to assess the robustness of the results [25].

Sensitivity analysis was executed after eliminating cases of spontaneous bacterial peritonitis. Subgroup evaluations were carried out based on several factors at ICU admission, including age, gender, site of infection, SOFA and MELD scores, the incidence of acute kidney injury, septic shock, platelet count, and the PaO2/FiO2 ratio. For continuous variables, the points of differentiation were obtained from either pre-existing knowledge or the interquartile range.

Second, we estimated the effectiveness of albumin administration using clinical parameters on the day of albumin infusion as indications, which the treating intensivists might have considered to decide whether albumin therapy should be initiated or not, including laboratory measurements (serum albumin concentration, serum lactate concentration), physiologic characteristics (MAP), and specific treatment regimens (NEE equivalent dose). Details about thresholds setting are shown in Additional file 1: Table S5. Schematic illustrations of the sub-cohort establishment are shown in Additional file 1: Figure S5. MSCMs were conducted in each sub-cohort to evaluate the impact of albumin administration on prognosis, weighted by adjusting for the confounding factors as aforementioned.

Third, the effects of albumin infusion were tested within different cutoffs of laboratory measurements. Initially, a data-driven threshold was determined by repeating the models with a 0.1 g/dL increase in the serum albumin threshold until reaching the highest value when albumin infusion was still associated with a reduction in risk of 28-day death. Then, another threshold was determined by repeating the models with a 0.1 mmol/L decrease in serum lactate threshold until reaching the lowest point when albumin infusion was consistently associated with decreased mortality.

Fourth, to further explore the effects of different albumin dosages on 28-day mortality, we stratified daily albumin exposure into five groups: no exposure, ≤ 0.5 g/kg, ≤ 1.0 and > 0.5 g/kg, ≤ 1.5 and > 1.0 g/kg, > 1.5 g/kg. Regression models were fitted using multinom (nnet) to calculate inverse probability weights of multi-groups [21]. We also assessed the treatment effects of different albumin solutions (5% and 25% concentrations) employing the same methodologies.

All statistical analyses were performed using R (version 4.2.2), and *P* < 0.05 was considered statistically significant. Bonferroni’s correction for multiple comparisons was used where appropriate.

## Results

### Patient characteristics

**Fig. 1:**
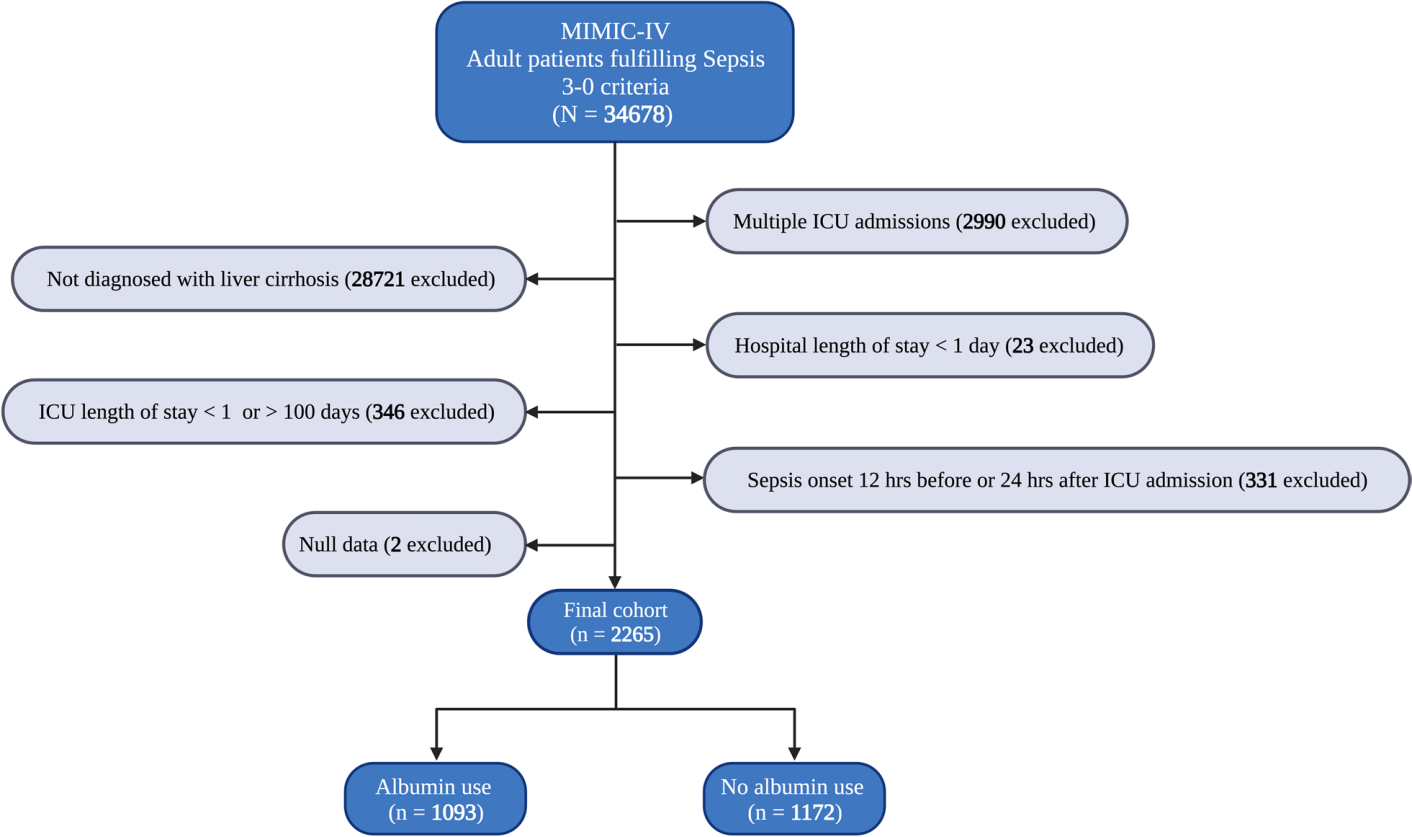
Flowchart showing patient selection for the study. MIMIC-IV: Medical Information Mart for Intensive Care IV; ICU: intensive care unit

A total of 2,265 septic patients with liver cirrhosis were included in the final analysis (Fig. 1). During the 28-day follow-up, 670 patients died (29.6%). Longitudinal data were collected for 12,441 ICU days. Out of this cohort, 1,093 patients (48.3%) received albumin infusion at least once during their ICU stay. Compared with the non-albumin group, patients in the albumin group were more severely ill, with a higher SOFA score (5 [IQR, 3–7] vs. 4 [IQR, 2–5]), a higher likelihood of receiving mechanical ventilation (662 [61%] vs. 647 [55%]), and a higher incidence of septic shock (375 [34%] vs. 182 [16%]). At ICU admission, the occurrence of acute kidney injury (AKI) was noticeably greater in the albumin-administered group compared to those not receiving albumin (854 [78%] vs. 619 [53%]). The group receiving albumin showed a significantly higher 28-day mortality rate than the group that did not (447 [41%] vs. 223 [19%]). Notably, there was no significant difference in serum albumin concentration at ICU admission between treated and untreated patients (Table 1).

**Table 1:** Baseline characteristics and outcomes of patients with and without IV albumin administration during ICU stay

Variables	Total patients (*n* = 2265)	Non-albumin group (*n* = 1172)	Albumin group (*n* = 1093)	*P* value
Male, [*n* (%)]	1453 (64)	773 (66)	680 (62)	0.07
Age, [years, M(IQR)]	59 (52, 67)	60 (52, 67)	58 (51, 66)	0.002
Weight, [kg, M(IQR)]	81 (69, 97)	80.45 (68, 96.3)	81.8 (69.2, 98.3)	0.126
Height, [cm, M(IQR)]	170 (165, 178)	173 (165, 178)	170 (163, 178)	0.185
Admission type^*^, [*n* (%)]				< 0.001
Elective	11 (0)	5 (0)	6 (1)	
Emergency	1420 (63)	784 (67)	636 (58)	
Observation	243 (11)	93 (8)	150 (14)	
Surgical	43 (2)	20 (2)	23 (2)	
Urgent	548 (24)	270 (23)	278 (25)	
*Infection site*, [*n* (%)]				< 0.001
Respiratory	400 (18)	167 (14)	233 (21)	
Gastrointestinal	173 (8)	62 (5)	111 (10)	
Genitourinary	186 (8)	101 (9)	85 (8)	
Others	1506 (66)	842 (72)	664 (61)	
*Severity of illness*				
CCI, [M(IQR)]	7 (5, 9)	7 (5, 9)	6 (5, 8)	0.138
APS III, [M(IQR)]	60 (45, 83)	51 (40, 68)	73 (55, 95)	< 0.001
OASIS, [M(IQR)]	35 (28, 42)	32 (26, 39)	37 (31, 44)	< 0.001
GCS, [M(IQR)]	15 (14, 15)	15 (14, 15)	15 (13, 15)	< 0.001
SOFA score, [M(IQR)]	4 (3, 6)	4 (2, 5)	5 (3, 7)	< 0.001
*Laboratory measurements*				
WBC, [10^9/L, M(IQR)]	10.7 (6.9, 16.2)	9.4 (6.2, 14.3)	12.2 (7.8, 18.3)	< 0.001
NLR, [M(IQR)]	8.8 (4.8, 16.0)	7.7 (4.3, 14.2)	9.5 (5.3, 17.4)	< 0.001
Chloride, [mmol/L, M(IQR)]	105 (100, 109)	106 (101, 110)	104 (99, 109)	< 0.001
Sodium, [mmol/L, M(IQR)]	138 (135, 142)	139 (135, 142)	138 (133, 141)	< 0.001
Hemoglobin, [g/dL, M(IQR)]	8.7 (7.5, 10.2)	9.1 (7.8, 10.6)	8.4 (7.2, 9.6)	< 0.001
ALT, [U/L, M(IQR)]	33 (21, 69)	33 (20, 65)	34 (21, 73)	0.06
AST, [U/L, M(IQR)]	71 (42, 146)	65 (38, 127)	78 (44, 170)	< 0.001
Albumin, [g/dL, M(IQR)]	2.8 (2.4, 3.2)	2.8 (2.4, 3.2)	2.8 (2.4, 3.3)	0.313
Total bilirubin, [mg/dL, M(IQR)]	2.7 (1.2, 6.6)	1.8 (0.9, 4)	4.4 (2, 9.7)	< 0.001
Platelet, [10^9/L, M(IQR)]	82 (52, 132)	88 (56, 145)	75 (48, 120)	< 0.001
INR, [M(IQR)]	1.7 (1.4, 2.3)	1.6 (1.3, 1.9)	2 (1.6, 2.6)	< 0.001
aPTT, [s, M(IQR)]	39.4 (33, 52.6)	35.7 (31, 44.7)	44.3 (36.1, 58.1)	< 0.001
Creatinine, [mg/dL, M(IQR)]	1.3 (0.9, 2.4)	1.1 (0.8, 2.1)	1.6 (1, 2.7)	< 0.001
BUN, [mmol/L, M(IQR)]	29 (18, 49)	26 (17, 43)	33 (20, 54)	< 0.001
BE, [mmol/L, M(IQR)]	− 2 (− 6, 0)	− 1 (− 5, 0)	− 4 (− 8, 0)	< 0.001
Bicarbonate, [mmol/L, M(IQR)]	20 (17, 23)	21 (18, 24)	19 (16, 22)	< 0.001
Lactate, [mmol/L, M(IQR)]	2.3 (1.6, 3.7)	2 (1.5, 3)	2.7 (1.8, 4.4)	< 0.001
PaO_2_, [mmHg, M(IQR)]	64 (43, 90)	67 (44, 94)	60 (42, 86)	< 0.001
PaCO_2_, [mmHg, M(IQR)]	41 (35, 48)	41 (35, 49)	41 (34, 48)	0.039
PaO_2_/FiO_2_ ratio [mmHg, M(IQR)]	185 (112.5, 272.5)	195 (123.25, 282.5)	176.67 (104, 262.5)	< 0.001
pH, [M(IQR)]	7.36 (7.28, 7.42)	7.37 (7.3, 7.42)	7.34 (7.26, 7.4)	< 0.001
*Vital signs*				
Heart rate, [beats ${min}^{-1}$, M(IQR)]	105 (91, 119)	104.5 (91, 118)	107 (93, 120)	0.016
MAP, [mmHg, M(IQR)]	56 (49, 63)	58 (51, 65)	55 (48, 61)	< 0.001
RR, [ breaths ${min}^{-1}$, M(IQR)]	27 (23, 32)	27 (23, 31)	27 (24, 32)	0.078
Temperature, [℃, M(IQR)]	37.2 (36.9, 37.6)	37.2 (36.9, 37.7)	37.1 (36.9, 37.6)	< 0.001
*Organ support components*				
Vasopressor use, [*n* (%)]	903 (40)	360 (31)	543 (50)	< 0.001
Vasopressor dose, [mcg/kg/min, M(IQR)]	0 (0, 0.15)	0 (0, 0.06)	0 (0, 0.24)	< 0.001
RRT, [*n* (%)]	161 (7)	74 (6)	87 (8)	0.149
Mechanical ventilation, [*n* (%)]	1309 (58)	647 (55)	662 (61)	0.011
Time to antibiotics, [hour, M(IQR)]	3.7 (1.8, 11.3)	3.8 (1.8, 10.8)	3.6 (1.8, 12.6)	0.965
Time to sepsis onset, [hour, M(IQR)]	− 1.9 (− 4.8, 0.6)	− 1.8 (− 4.7, 0.6)	− 2.0 (− 4.8, 0.5)	0.066
Urine output, [mL, M(IQR)]	1155 (588, 1853)	1375 (785, 2102.5)	905 (450, 1575)	< 0.001
Fluid balance, [mL, M(IQR)]	437 (− 951, 1909)	93 (− 1198, 1376)	847 (− 536, 2426)	< 0.001
Spontaneous bacterial peritonitis, [*n* (%)]	186 (8)	33 (3)	153 (14)	< 0.001
Septic shock, [*n* (%)]	557 (25)	182 (16)	375 (34)	< 0.001
Acute kidney injury, [*n* (%)]	1473 (65)	619 (53)	854 (78)	< 0.001
ICU readmission, [*n* (%)]	178 (8)	67 (6)	111 (10)	< 0.001
28-day mortality, [*n* (%)]	670 (30)	223 (19)	447 (41)	< 0.001
90-day mortality, [*n* (%)]	875 (39)	314 (27)	561 (51)	< 0.001
Hospital mortality, [*n* (%)]	573 (25)	181 (15)	392 (36)	< 0.001
Hospital-free days, [days, M(IQR)]	10 (0, 21)	18 (0, 23)	0 (0, 15)	< 0.001
ICU-free days, [days, M(IQR)]	23.1 (0, 25.9)	25.0 (19.0, 26.2)	15.2 (0, 24.6)	< 0.001

CCI = Charlson Comorbidity Index; SAPS II = Simplified Acute Physiology Score II; OASIS = Oxford Acute Severity of Illness Score; SOFA = Sequential Organ Failure Assessment; WBC = white blood cells; NLR = neutrophil-to-lymphocyte ratio; ALT = alanine transaminase; AST = aspartate aminotransferase; GCS = Glasgow Coma Scale; INR = international normalized ratio; aPTT = activated partial thromboplastin clotting time; BUN = blood urea nitrogen; MAP = mean arterial pressure; BE = base excess; PaCO_2_ = partial pressure of carbon dioxide; PaO_2_/FiO_2_ ratio = ratio of arterial oxygen partial pressure to fractional inspired oxygen; RR = respiration rate; RRT = renal replacement therapy; ICU = intensive care unit

*The 'AMBULATORY OBSERVATION,' 'DIRECT OBSERVATION,' 'EU OBSERVATION,' and 'OBSERVATION ADMIT' categories were consolidated into a single group labeled ‘observation.’

Vital signs, laboratory measurements, and other time-varying variables presented in this table were collected within 24 h of ICU admission. If a variable was recorded more than once in 24 h, we used the value related to the greatest severity of illness

**Fig. 2:**
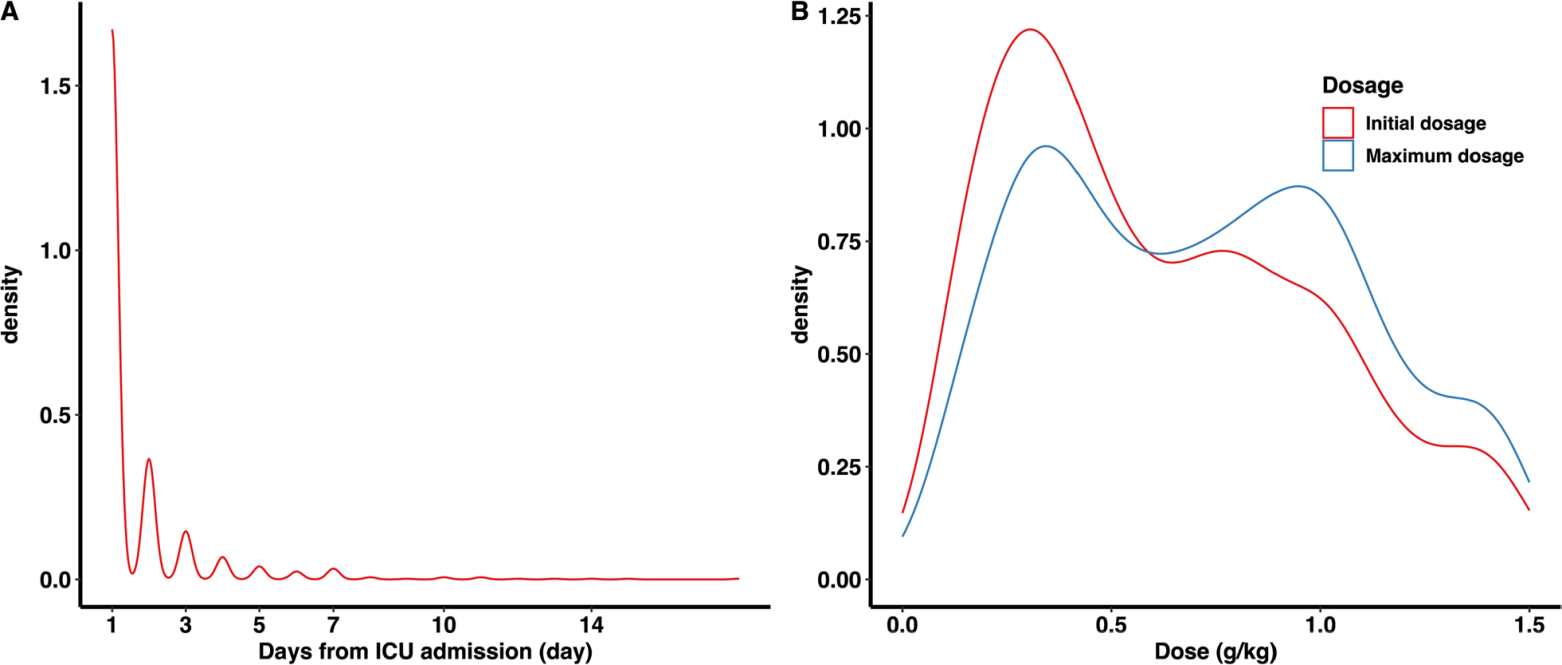
Distribution of time to initiate albumin administration (**A**) and dosage of albumin therapy (**B**)

Albumin administration had a median initiation time of 1.0 days (IQR 1.0–2.0) from ICU admission. The initial and maximum daily doses administered were 0.62 g/kg (IQR 0.32–1.03) and 0.82 g/kg (IQR 0.42–1.19), respectively. The duration of albumin treatment was 2.0 days (IQR, 1.0–3.0 days), with a maximum of 25.0 days. Figure 2 shows the time distribution from ICU admission to initiation of albumin administration (Fig. 2A) and the initial and maximum daily dosage of albumin therapy (Fig. 2B).

### Impact of albumin administration on the risk of 28-day death

According to the results of the marginal structure Cox proportional model, albumin administration was associated with a reduced probability of death across the 28-day follow-up (HR 0.76 [95% CI 0.61–0.94]) (Table 2) (Additional file 1: Figure S6). The distributions of IPW in albumin and non-albumin groups are shown in Additional file 1: Figure S7. However, using Heaviside functions, this association remained significant only in the first week, when evaluated by each week during the 28 days. The results of the multivariate MSCM in the overall population were in accordance with the univariate MSCM (HR 0.64 [95% CI 0.51–0.80]) (Additional file 1: Table S6). Besides, the association was confirmed by the extended Cox proportional hazards model (HR 0.74 [95% CI 0.61–0.89]) (Additional file 1: Table S7).

**Table 2:** MSCM for the effect of albumin administration with the trend over the follow-up on 28-day mortality

Marginal structural cox proportional hazards model for 28-day mortality						
Variable	Overall effect during follow-up	*P* value	Heaviside step functions			
			Week 1	Week 2	Week 3	Week 4
Albumin administration	0.76 (0.61–0.94)	0.0132	0.72 (0.56–0.92)*	0.79 (0.47–1.32)	1.08 (0.35–3.30)	2.52 (0.42–14.96)

MSCM = Marginal Structural Cox proportional hazards Model

### Sensitivity analysis

After the exclusion of patients with spontaneous bacterial peritonitis, 2,079 patients remained. The results of the sensitivity analysis were consistent with the primary analysis: Weighted by MSCM, albumin infusion was associated with reduced 28-day mortality regardless of the univariate (HR 0.72 [95% CI 0.56–0.92]) or multivariate model (HR 0.62 [95% CI 0.49–0.80]) (Additional file 1: Table S8).

### Subgroup analysis

Albumin administration was associated with lower 28-day mortality in patients with MELD score ≥ 20 (HR 0·68 [95% CI 0·54–0·84]), septic shock (HR 0·65 [95% CI 0·48–0·89]), and total bilirubin ≥ 3.0 mg/dl (HR 0.61 [95% CI 0·47–0·79]), while no interaction was detected. More importantly, significant interaction relationships were observed among patients with acute kidney injury (HR 0.66 [95% CI 0·52–0·83] vs. HR 0.99 [95% CI 0·59–1.67]; *P* for interaction = 0.019) and low platelet counts (HR 0.59 [95% CI 0·41–0·83] vs. HR 0.94 [95% CI 0·71–1.25]; *P* for interaction = 0.018). However, no such interactive effects were found between albumin administration and different infection sites regarding 28-day mortality (Additional file 1: Figure S8).

### Indications for albumin administration

**Fig. 3:**
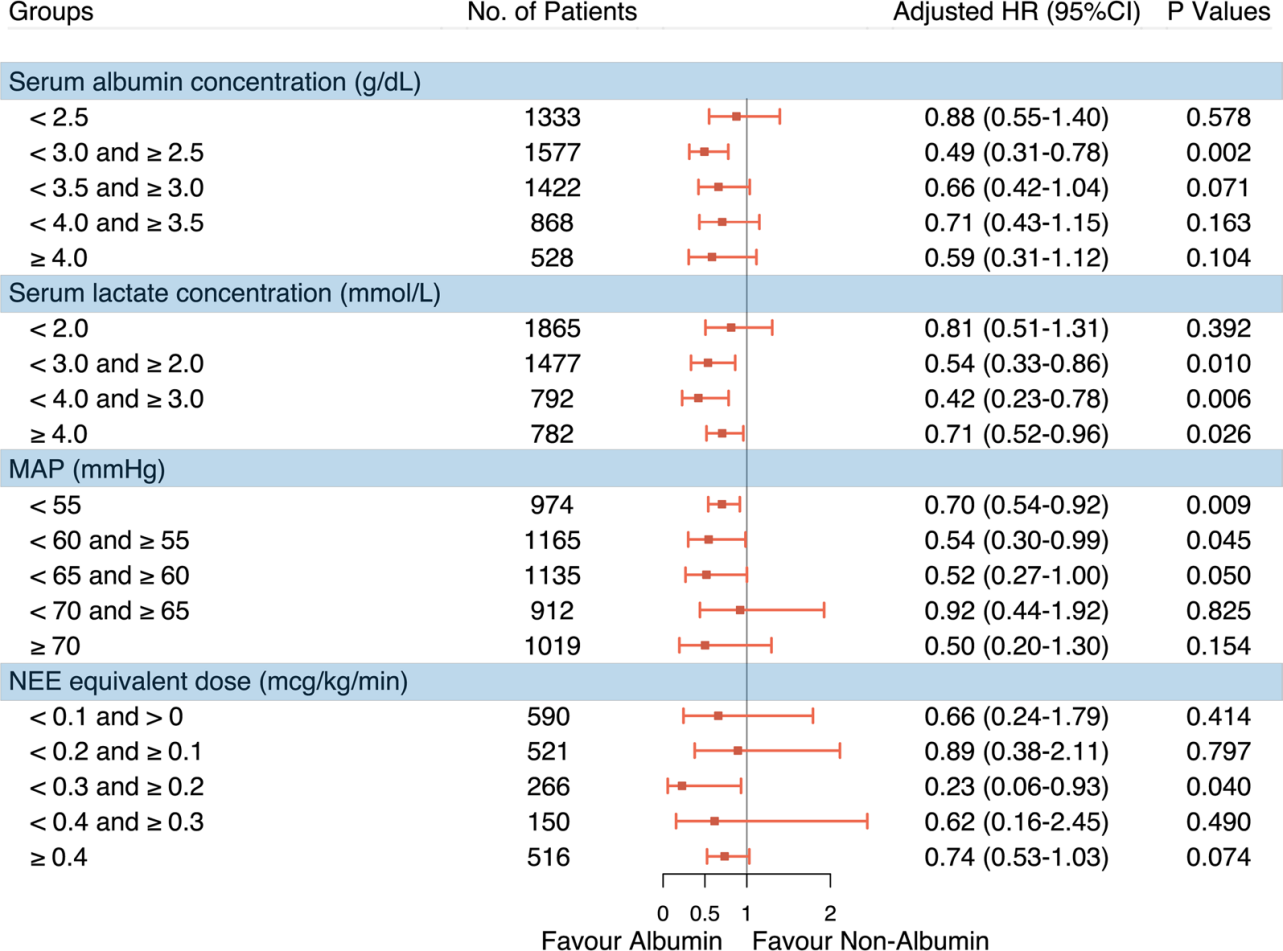
Sub-cohort analyses of the association between albumin administration and 28-day mortality using MSCM. HR: hazard ratio; NEE: norepinephrine equivalence

The effects of albumin administration were accessed under varying serum albumin concentrations (Fig. 3). We detected that albumin therapy significantly improved outcomes only when initiated in patients with a serum albumin concentration of 2.5–3.0 g/dL, with a hazard ratio for 28-day mortality of 0.49 (0.31 to 0.78). The following hazard ratios for mortality were measured if albumin administration had been initiated according to the other four prespecified thresholds for serum albumin concentration: < 2.5 g/dL, 0.88 (0.55 to 1.40); < 3.5 and ≥ 3.0 g/dL, 0.66 (0.42 to 1.04); < 4.0 and ≥ 3.5 g/dL, 0.71 (0.43 to 1.15); and ≥ 4.0 g/dL, 0.59 (0.31–1.12). Additionally, the highest serum albumin concentration derived as a trigger for albumin administration associated with a reduced risk of death was 2.7 g/dL (Additional file 1: Table S9).

Likewise, when comparing albumin treatment strategies initiated in patients with higher time-dependent serum lactate concentrations to those not receiving albumin treatment, the results suggested albumin therapy significantly improved outcomes only when initiated in patients with a serum lactate concentration of more than or equal to 2.0 mmol/L. Subsequently, we identified that the lowest serum lactate concentration derived as a trigger for albumin administration associated with a reduced risk of death was 2.2 mmol/L (Additional file 1: Table S10). We also observed that albumin therapy significantly increased 28-day survival only when initiated in patients with a MAP of less than 60 mmHg, with a hazard ratio of 0.54 (0.30 to 0.99), regardless of the vasopressor dose. Further, for patients receiving vasopressor regimens, it is optimal to initiate albumin administration when the NEE equivalent dose is between 0.2 and 0.3 mcg/kg/min.

### Secondary analysis

Adopting multinomial MSCM weighted for the same covariates as above, the association between different albumin dosages and 28-day mortality was demonstrated. The results revealed that the beneficial effects of albumin infusion were compromised when the daily dose exceeded 1.0 g/kg (daily dose ≤ 1.5 and > 1.0 g/kg: HR 0.81 [95% CI 0.50–1.29]) (Table 3). Additionally, our analysis indicated that both 25% and 5% albumin solution administrations were associated with reduced 28-day mortality (Additional file 1: Table S11).

**Table 3:** Association between different albumin dosage strata and 28-day mortality estimated by the MSCM

Albumin dosage^*^ strata	No. of patients	Hazard ratio	Lower limit of 95% CI	Upper limit of 95% CI	*P* value
≤ 0.5 g/kg	665	0.62	0.43	0.88	0.008
≤ 1.0 and > 0.5 g/kg	547	0.52	0.34	0.80	0.003
≤ 1.5 and > 1.0 g/kg	299	0.81	0.50	1.29	0.37
> 1.5 g/kg	151	0.87	0.39	1.94	0.73

*The albumin dosage was calculated on a daily basis using the formula: Albumin dosage = [amount ^A^ (mL) × concentration ^A^ + amount ^B^ (mL) × concentration ^B^ + …]/weight (kg)

CI = Confidence interval; MSCM = Marginal Structural Cox proportional hazards Model

## Discussion

Our study on cirrhotic patients with sepsis has many novel findings that merit further discussion. First, dynamic albumin infusion was associated with a reduced 28-day risk of death, especially during the first week of illness, but only in specific sub-cohorts. Second, in patients when albumin administration was initiated on the day of serum albumin concentration between 2.5 and 3.0 g/dL, serum lactate concentration greater than or equal to 2 mmol/L, MAP less than 60 mmHg, or vasopressor dosage between 0.2 and 0.3 mcg/kg/min (norepinephrine equivalent, NEE), we found that albumin infusion would have been most effective.

Previous studies proved that albumin administration could significantly improve the hemodynamics of septic patients with cirrhosis as a resuscitation fluid, while the sustained benefit of albumin administration was only identified in septic shock in a subgroup analysis of the SAFE study [26]. Our data confirmed the beneficial effects of daily albumin administration in septic patients with cirrhosis, and baseline subgroups suggested that patients with higher severity of illness and more organ dysfunctions would benefit more from albumin infusion. The oncotic properties of albumin are well known. Moreover, human serum albumin displays pivotal secondary functions in patients with cirrhosis, including antioxidant, immune-modulating effects, and positive inotropic effects, which could explain the longitudinal association between albumin infusion and prognosis.

Limited data were available regarding the indications for albumin infusion in septic patients with cirrhosis. Hypoalbuminemia (generally defined as a serum albumin concentration ≤ 30 g/L) was a prognostic biomarker in acutely ill patients, and each 10 g/L decrease in serum albumin concentration was associated with a 137% increase in the odds of death, an 89% increase in morbidity, and a 71% increase in length of hospital stay [11]. Correction of hypoalbuminemia via albumin infusion remains controversial. A pilot study found that maintaining serum albumin of more than 30 g per liter results in improved organ function, a less positive fluid balance, and a better tolerance of enteral feeding in critically ill patients [10]. A meta-analysis of dose-dependency in controlled trials of albumin therapy hypothesized that complication rates might be reduced when the serum albumin level attained during albumin administration exceeds 30 g/L, while no significant survival benefit was demonstrated [11]. The ALBIOS study also declared that in patients with severe sepsis, daily administration of albumin to maintain a serum albumin level of 30 g per liter or more, compared with crystalloids alone, did not improve the survival rate at 28 and 90 days [12]. Our analysis generated a reliable, data-driven threshold for serum albumin concentration at 2.7 g/dL, beneath which the albumin administration corresponded to a diminished 28-day mortality risk. A closer examination of specific sub-cohorts derived from real-time clinical parameters revealed that the employment of albumin was particularly beneficial when commenced in septic patients with liver cirrhosis, exhibiting serum albumin levels between 2.5 and 3.0 g/dL. Nevertheless, we also noted that those with a serum albumin concentration of less than 2.5 g/dL did not experience the same advantageous effects from albumin infusion. The observed divergence can likely be attributed to the circumstance that this specific sub-cohort was not enduring a continuous state of illness—during which the proposed treatment might significantly impact outcomes—but was rather facing a rapid mortality scenario [27]. Furthermore, despite our rigorous efforts to adjust for potential biases, it is possible that some residual confounders were not fully eliminated.

Meanwhile, we also explored the potential indications for albumin infusion except for hypoalbuminemia and declared that albumin infusion would also be effective if provided to selected patients with unstable hemodynamic status, indicating by serum lactate greater than or equal to 2 mmol/L, MAP less than 60 mmHg, or vasopressor dosage between 0.2 and 0.3 mcg/kg/min (NEE), regardless of the serum albumin concentration. Although the beneficial effects of albumin infusion on hemodynamic status have been proved, no clear criteria were available. The present study identified clear criteria using a data-driven approach under real-world conditions.

The systemic and organ inflammation-modulating effect of albumin highly depends on the post-treatment serum albumin concentration, emphasizing the importance of albumin dosage [28, 29]. The three randomized controlled trials so far published assessing the beneficial effects of albumin therapy in cirrhosis patients with non-SBP infection draw similar conclusions that receiving albumin 1.5 g/kg on day 1 and 1 g/kg on day 3 would not improve survival [30–32]. A more troublesome observation was that Thévenot et al. [32] found nine patients who suffered from pulmonary edema following albumin infusion, two of whom died within a short time window after albumin infusion. These findings were validated in our study. The association between different albumin dosages and 28-day mortality suggested that the therapeutic effects of albumin infusion were compromised when the daily dose exceeded 1.0 g/kg. Despite the potential benefits of albumin, its protective oncotic effect may be diminished when capillary permeability increases, a condition well established in cirrhotic patients with sepsis, exacerbated by impaired lymphatic circulation [33–35]. Increased extravasation of albumin from capillaries may result in accumulation within the extravascular spaces, leading to fluid overload subsequently [36]. Overall, from a clinical point of view, we suggested that a large amount of albumin administration should be avoided in patients with prominent pulmonary capillary permeability alterations, or given cautiously after a thorough assessment of cardiac function and volume status.

Several limitations in the present study should be acknowledged. First, this study was based on electronic healthcare records of routine clinical practice with missing data and outliers. To retain statistical power, we used multiple imputation by weighted predictive mean matching to reduce the risk of bias from missing data. Second, the single-database design requires further validation to confirm its beneficial effects. Third, it is imperative to acknowledge that the MIMIC-IV database collects data over a time span of more than a decade. Consequently, our findings might be influenced by changes in the guidelines for sepsis and liver cirrhosis that occurred during this period, which could affect the practical relevance of our results. Fourth, this study included patients who belong to a well-defined and characterized clinical entity. Thus, the threshold may not apply to other patients. Fifth, whether the benefits we observed are primarily attributed to sepsis management, cirrhosis-related complications, or an amalgamation of both remains an essential question that needs to be addressed. The therapeutic effects of albumin between septic patients with and without liver cirrhosis warrant further investigation.

## Conclusion

In conclusion, dynamic albumin administration provided a significant survival benefit at 28 days in septic patients with liver cirrhosis, especially in specific sub-cohorts. Serum albumin concentration, serum lactate, MAP, and vasopressor dosage were found to be modifiers of treatment effectiveness and should be considered when deciding to initial albumin infusion. Large amounts of albumin infusion (> 1.0 g/kg) should be cautiously administered in patients with prominent pulmonary capillary permeability alterations. Prospective interventional trials are needed to confirm these findings.

## Supplementary Information

**Additional file 1: Additional method. Table S1:** Selection strategy for variables with multiple measurements. **Table S2:** Infection category codes from ICD-9-CM to ICD-10-CM. **Table S3**: Missing rate for demographics and clinical variables extracted from the database during the observation period. **Table S4:** Missing rate for demographics and clinical variables extracted from the database on the first day. **Table S5:** Thresholds for clinical variables as albumin therapy indication. **Table S6:** Multivariable Cox model with time-fixed and time-dependent covariates for 28-day mortality adding to the model the effect of albumin administration, weighted with marginal structural models. **Table S7:** Association between albumin administration and 28-day mortality estimated by extended Cox regression model with time-varying covariates. **Table S8**: Univariate and multivariate analyses weighted with MSCM of the association between albumin infusion and 28-day mortality in patients after the exclusion of spontaneous bacterial peritonitis. **Table S9**: Multivariable Cox model with time-fixed and time-dependent covariates for 28-day mortality adding to the model the effect of albumin administration, weighted with marginal structural models. The analysis was repeated until reaching the highest level of serum albumin concentration, for which the albumin infusion still showed a positive effect on the outcome. **Table S10**: Multivariable Cox model with time-fixed and time-dependent covariates for 28-day mortality adding to the model the effect of albumin administration, weighted with marginal structural models. The analysis was repeated until reaching the lowest level of serum lactate concentration, for which the albumin infusion still showed a positive effect on the outcome. **Table S11**: Correlation between the types of albumin solutions administered and 28-day mortality as estimated by the MSCM. **Figure S1:** Directed acyclic graph (DAG) illustrating the potential actions of confounding covariates on the relation between the administration of albumin according to different albumin concentrations and clinical outcomes. **Figure S2:** Correlation matrix for time-fixed covariates. **Figure S3:** Correlation matrix for time-dependent covariates. **Figure S4:** Variable selection for Cox time-dependent and time-independent model factors associated with 28-day mortality. **Figure S5:** Schematic illustrations of the sub-cohort establishment. **Figure S6:** Expanded Kaplan–Meier survival analysis, weighted by marginal structural models. **Figure S7:** Distribution of inverse probability weight in albumin and non-albumin groups. **Figure S8: **Adjusted risk of death at 28 days measured by Marginal Structural Cox proportional hazards Model according to baseline subgroups.

## Data Availability

Data are available on reasonable request.

## Abbreviations

SBP: Spontaneous bacterial peritonitis
MELD: Model for End-Stage Liver Disease
HAS: Human albumin solution
MSCM: Marginal Structural Cox proportional hazards Model
IPW: Inverse probability weighting
EHR: Electronic healthcare record
GCS: Glasgow Coma Scale
HR: Hazard ratio
CI: Confidence interval
ICD: International Classification of Diseases
ICU: Intensive care unit
IQR: Interquartile range
IRB: Institutional review board
MIMIC-IV: Medical Information Mart for Intensive Care IV
PaCO_2_: Partial pressure of carbon dioxide
PaO_2_: Arterial partial oxygen pressure
RRT: Renal replacement therapy
SOFA: Sequential Organ Failure Assessment
MICE: Multiple imputations with chained equations
