# Supplementary material for: Identification of indications for albumin administration in septic patients with liver cirrhosis
Source: Crit Care. 2023 Jul 28;27:300. doi: 10.1186/s13054-023-04587-3 (PMC10385943; doi:10.1186/s13054-023-04587-3)
Supplement: Supplementary file 1 — Additional file 1: Additional method. Table S1: Selection strategy for variables with multiple measurements. Table S2: Infection category codes from ICD-9-CM to ICD-10-CM. Table S3: Missing rate for demographics and clinical variables extracted from the database during the observation period. Table S4: Missing rate for demographics and clinical variables extracted from the database on the first day. Table S5: Thresholds for clinical variables as albumin therapy indication. Table S6: Multivariable Cox model with time-fixed and time-dependent covariates for 28-day mortality adding to the model the effect of albumin administration, weighted with marginal structural models. Table S7: Association between albumin administration and 28-day mortality estimated by extended Cox regression model with time-varying covariates. Table S8: Univariate and multivariate analyses weighted with MSCM of the association between albumin infusion and 28-day mortality in patients after the exclusion of spontaneous bacterial peritonitis. Table S9: Multivariable Cox model with time-fixed and time-dependent covariates for 28-day mortality adding to the model the effect of albumin administration, weighted with marginal structural models. The analysis was repeated until reaching the highest level of serum albumin concentration, for which the albumin infusion still showed a positive effect on the outcome. Table S10: Multivariable Cox model with time-fixed and time-dependent covariates for 28-day mortality adding to the model the effect of albumin administration, weighted with marginal structural models. The analysis was repeated until reaching the lowest level of serum lactate concentration, for which the albumin infusion still showed a positive effect on the outcome. Table S11: Correlation between the types of albumin solutions administered and 28-day mortality as estimated by the MSCM. Figure S1: Directed acyclic graph (DAG) illustrating the potential actions of confounding covariates on the rel [file 13054_2023_4587_MOESM1_ESM.docx]

**Supplemental Digital Content for the study entitled:** **Identification of Indications for Albumin Administration in Septic Patients with Liver Cirrhosis**

**Additional methods:**

1. **Definition of “Sepsis”**

Sepsis was defined based on previously described methods, including (1) Clinical suspicion of infection as determined by the earlier timestamp of antibiotics administration, and cultures within a certain timeframe. If antibiotics were given first, then the cultures must have been obtained within 24 hours. If cultures were obtained first, then antibiotics must have been subsequently ordered within 72 hours. (2) The occurrence of end-organ damage as identified by a two-point deterioration in SOFA score. (3) The onset time of sepsis is the earlier of $t_{\mathrm{suspicion}}$ and $t_{\mathrm{SOFA}}$ as long as $t_{\mathrm{SOFA}}$ occurs no more than 48 hours before or 24 hours after $t_{\mathrm{suspicion}}$; otherwise, the patient is not marked as a sepsis patient. Specifically, if $t_{\mathrm{suspicion}}$ -48 ≤ $t_{\mathrm{SOFA}}$ ≤ $t_{\mathrm{suspicion}}$ +24, then $t_{\mathrm{sepsis}}$ = min ($t_{\mathrm{suspicion}}$, $t_{\mathrm{SOFA}}$) [1, 2]. The Structured Query Language (SQL) code used for data extraction can be found at [mimic-iv/concepts/sepsis at master · MIT-LCP/mimic-iv · GitHub](https://github.com/MIT-LCP/mimic-iv/tree/master/concepts/sepsis).

1. **Approach to missing data**

Missing data were summarized in Tables S3 and S4. No consensus exists regarding the standard percentage of missing values for excluding a variable from analysis. For our study, we have elected to set our threshold at 60%, bearing in mind that Zhang et al. [3] have omitted variables with over 70% missing values in their analysis. Before each model fitting process, we assumed missing data were “missing at random” (MAR) [4, 5]. Multiple imputation by chained equation (MICE), which generates values for all missing data using the observed data for all patients, was adopted. The imputation method was weighted predictive mean matching. The *“mice”* package in R studio was employed to impute the data [6].

1. **Marginal structural COX model**

Marginal structural Cox models (MSCM) provide the marginal causal relation between a time-varying exposure (albumin infusion) and a survival outcome (e.g., death at day 28), controlling for time-varying confounders (SOFA score, serum lactate concentration, PaO_2_/FiO_2_ ratio, vasopressor dose, Daily UO, and MAP) without conditioning on those variables. Since the treatment with albumin administration might change during the follow-up period, this was considered a time-dependent variable. Potential confounders such as age, gender, admission type, ethnicity, infection site, and serum albumin concentration were obtained on day 1 after ICU admission.

The parameters of MSCM could be estimated using inverse probability weighting (IPW) to correct both confounding and forms of selection bias, such as informative censoring [7]. Two pseudo-populations are created by weighting each patient using IPW, similar to baseline and time-dependent confounding factors, and different in albumin treatment.

The MSCM model allows the comparison of hazard functions for patients who never received albumin infusion with those who received albumin treatment [8]. The partial likelihood function of the Cox model was modified such that the contribution of patient $i$ to the risk set was weighted by the stabilized inverse probability of treatment and censoring (IPTC) weight, $\mathrm{sw}_{\mathrm{it}}$, to account for potential confounding effects induced by baseline (i.e., variables obtained on day 1 including age, gender, admission type, ethnicity, infection site, and serum albumin concentration) and time-varying confounders (i.e., SOFA score, serum lactate concentration, PaO_2_/FiO_2_ ratio, vasopressor dose, Daily UO, and MAP). The stabilized inverse probability of treatment (IPT) for patient $i$ at day t can be obtained by

$$\mathrm{sw}_{\mathrm{it}}^{T}=\prod_{j=0}^{t} \frac{\mathrm{pr}\left( A_{\mathrm{ij}}=a_{\mathrm{ij}}|\bar{A}_{i\left( j-1 \right)}=\bar{a}_{i\left( j-1 \right)},L_{i0}=l_{i0} \right)}{\mathrm{pr}\left( A_{\mathrm{ij}}=a_{\mathrm{ij}}|\bar{A}_{i\left( j-1 \right)}=\bar{a}_{i\left( j-1 \right)},L_{i0}=l_{i0},\bar{L}_{\mathrm{ij}}=\bar{l}_{\mathrm{ij}} \right)}$$

Where $A_{\mathrm{ij}}$ represents the treatment with albumin for patient i in day j. $L_{i0}$ and $L_{\mathrm{ij}}$are the baseline and time-varying variables, respectively, for patient i. $\bar{A}_{i\left( j \right)}=\bar{a}_{i\left( j \right)}$, and $\bar{L}_{\mathrm{ij}}=\bar{l}_{\mathrm{ij}}$ are observed treatment and time-varying confounder history, respectively, from baseline to time j. Since the time-varying confounders appeared only in the denominator of the weights, the stabilized weights were inversely related to a function of the time-varying confounder cumulative relapse. The weights $\mathrm{sw}_{\mathrm{it}}^{T}$ down-weighted the person-day contributions when cumulative time-varying confounders were a strong predictor of treatment status in the subsequent time periods, after controlling for the baseline covariates. The weights created a pseudo-population in which SOFA score, serum lactate concentration, PaO_2_/FiO_2_ ratio, vasopressor dose, Daily UO, and MAP no longer predicted subsequent albumin treatment status. The estimates of the effect of albumin treatment in this pseudo-population would be an unbiased estimate for the target population. Similarly, the inverse probability of censoring can be estimated in the same way:

$$\mathrm{sw}_{\mathrm{it}}^{C}=\prod_{j=0}^{t} \frac{\mathrm{pr}\left( C_{\mathrm{ij}}=0|\bar{C}_{i\left( j-1 \right)}=0,\bar{A}_{i\left( j-1 \right)}=\bar{a}_{i\left( j-1 \right)},L_{i0}=l_{i0} \right)}{\mathrm{pr}\left( C_{\mathrm{ij}}=0|\bar{C}_{i\left( j-1 \right)}=0,\bar{A}_{i\left( j-1 \right)}=\bar{a}_{i\left( j-1 \right)},L_{i0}=l_{i0},\bar{L}_{i(j-1)}=\bar{l}_{i(j-1)} \right)}$$

Then the overall stabilized IPTW weights were obtained by the product of $\mathrm{sw}_{\mathrm{it}}=\mathrm{sw}_{\mathrm{it}}^{C}\times\mathrm{sw}_{\mathrm{it}}^{T}$. Finally, an MSCM was fit using a robust variance estimator, and time-varying treatment assignment was included in the model [9]. The *“ipw”* package was employed for the MSCM analysis [10].

**Reference:**

1. Yang M, Liu C, Wang X, Li Y, Gao H, Liu X, Li J: **An Explainable Artificial Intelligence Predictor for Early Detection of Sepsis**. *Crit Care Med* 2020, **48**(11):e1091-e1096.

2. Reyna MA, Josef CS, Jeter R, Shashikumar SP, Westover MB, Nemati S, Clifford GD, Sharma A: **Early Prediction of Sepsis From Clinical Data: The PhysioNet/Computing in Cardiology Challenge 2019**. *Crit Care Med* 2020, **48**(2):210-217.

3. Zhang Z, Ho KM, Hong Y: **Machine learning for the prediction of volume responsiveness in patients with oliguric acute kidney injury in critical care**. *Crit Care* 2019, **23**(1):112.

4. Montez-Rath ME, Winkelmayer WC, Desai M: **Addressing missing data in clinical studies of kidney diseases**. *Clin J Am Soc Nephrol* 2014, **9**(7):1328-1335.

5. Dziura JD, Post LA, Zhao Q, Fu Z, Peduzzi P: **Strategies for dealing with missing data in clinical trials: from design to analysis**. *Yale J Biol Med* 2013, **86**(3):343-358.

6. Zhang Z: **Multiple imputation with multivariate imputation by chained equation (MICE) package**. *Ann Transl Med* 2016, **4**(2):30.

7. Robins JM, Hernan MA, Brumback B: **Marginal structural models and causal inference in epidemiology**. *Epidemiology* 2000, **11**(5):550-560.

8. Karim ME, Gustafson P, Petkau J, Zhao YS, Shirani A, Kingwell E, Evans C, van der Kop M, Oger J, Tremlett H: **Marginal Structural Cox Models for Estimating the Association Between beta-Interferon Exposure and Disease Progression in a Multiple Sclerosis Cohort**. *American Journal of Epidemiology* 2014, **180**(2):160-171.

9. Hernan MA, Brumback B, Robins JM: **Marginal structural models to estimate the causal effect of zidovudine on the survival of HIV-positive men**. *Epidemiology* 2000, **11**(5):561-570.

10. van der Wal WM, Geskus RB: **ipw: An R Package for Inverse Probability Weighting**. *Journal of Statistical Software* 2011, **43**(13):1-23.

**Table S1: Selection strategy for variables with multiple measurements.**

| Data items | Details |
| --- | --- |
| GCS | Record the **lowest** value for 24 hours of ICU admission |
| WBC | Record the **highest** value for 24 hours of ICU admission |
| NLR | Record the **highest** value for 24 hours of ICU admission |
| Chloride | Record the **highest** value for 24 hours of ICU admission |
| Sodium | Record the **highest** value for 24 hours of ICU admission |
| Hemoglobin | Record the **lowest** value for 24 hours of ICU admission |
| ALT | Record the **highest** value for 24 hours of ICU admission |
| AST | Record the **highest** value for 24 hours of ICU admission |
| Albumin | Record the **lowest** value for 24 hours of ICU admission |
| Total bilirubin | Record the **highest** value for 24 hours of ICU admission |
| Platelet | Record the **lowest** value for 24 hours of ICU admission |
| INR | Record the **highest** value for 24 hours of ICU admission |
| aPTT | Record the **highest** value for 24 hours of ICU admission |
| Creatinine | Record the **highest** value for 24 hours of ICU admission |
| BUN | Record the **highest** value for 24 hours of ICU admission |
| BE | Record the **lowest** value for 24 hours of ICU admission |
| Bicarbonate | Record the **lowest** value for 24 hours of ICU admission |
| Lactate | Record the **highest** value for 24 hours of ICU admission |
| PaCO_2_ | Record the **highest** value for 24 hours of ICU admission |
| PaO_2_/FiO_2_ ratio | Record the **lowest** value for 24 hours of ICU admission |
| pH | Record the **lowest** value for 24 hours of ICU admission |
| Heart rate | Record the **highest** value for 24 hours of ICU admission |
| MAP | Record the **lowest** value for 24 hours of ICU admission |
| RR | Record the **highest** value for 24 hours of ICU admission |
| Temperature | Record the **highest** value for 24 hours of ICU admission |
| Vasopressor dose | Record the **highest** value for 24 hours of ICU admission |

WBC = White blood cells; NLR = Neutrophil to lymphocyte ratio; ALT = Alanine transaminase; AST = aspartate aminotransferase; GCS = Glasgow Coma Scale; INR = International normalized ratio; aPTT = activated Partial Thromboplastin Clotting Time; BUN = Blood Urea Nitrogen; MAP = Mean Arterial Pressure; BE = Base excess; PaCO2 = Partial Pressure of Carbon Dioxide; PaO2/FiO2 ratio= Ratio of arterial oxygen partial pressure to fractional inspired oxygen; RR = Respiration rate.

**Table S2: Infection category codes from ICD-9-CM to ICD-10-CM.**

| **Category** | | | **ICD-9-CM codes** | **ICD-10-codes** |
| --- | --- | --- | --- | --- |
| **Lung infection** | | | "010","011","012","0310","481","482","485","486" | "A157","A156","A155","A154","A158","A310","A311","A312","A318","A319","J13","J181","J150","J151","J14","J154","J153","J1520","J15211","J15212","J1529","J158","J155","J156","A481","J180","J189" |
| **Gastrointestinal infection** | **Peritoneal infection** | | "00845","567","56983" | "A0471","A0472","K67","K658","K650","K651","K652","K650","K6812","K6819","K689","K653","K654","K658","K631" |
|  | **Abdominal infection** | | "003","014","540","541","542","56201","56203","56211","56213","566","5695","5720","5721","5750" | "A020","A0220","A0229","A028","A029","A1831","A1832","A1839","K352","K353","K3580","K3589","K37","K36","K5712","K5713","K5732","K5733","K610","K611","K613","K630","K750","K751","K810" |
| **Genito-urinary infection** | | | "016","098","590","597","5990","601","614","615","616" | "A1811”,”A1810”,”A1812”,”A1813”,”A1815”,”A1814”,”A1817”,”A1816”,”A1818”,”A5400”,”A5429”,”A5401","A5422”,”A5423”,”A5403”,”A5424”,”A5421”,”A5442”,”N110”,”N151”,”N340”,”N341”,”N342”,”N343”,”N390","N410”,”N411”,”N412”,”N413”,”N51”,”N414”,”N418”,”N419”,”N7001”,”N7002”,”N7003”,”N7011”,”N7012","N7013”,”N7091”,”N7092”,”N7093”,”N730”,”N731”,”N732”,”N733”,”N736”,”N734”,”N738”,”N739”,”N710","N711”,”N719”,”N72”,”N760”,”N761”,”N762”,”N763”,”N771”,”N750”,”N751”,”N764”,”N766”,”N770”,”N7681","N759”,”N765”,”N7689" |
| **Other infection** | | **Septicemia** | "038","78552","99592" | "A409","A412","A4101","A4102","A411","A403","A414","A4150","A413","A4151","A4152","A4153","A4159","A4189","A419","R6521","R6520" |
|  |  | **Heart infection** | "420","421" | "I32","I309","I300","I308","I330","I39","I339" |
|  |  | **Fungal infection** | "1120","1124","1125","114","115","116","117","118" | "B370","B3783","B371","B377","B380","B383","B384","B3889","B381","B382","B389","B394","G02","H32","B392","B393","B395","J17","B399","B409","B410","B419","B480","B481","B420","B421","B427","B429","B439","B449","B470","B450","B457","B459","B482","B469","B488","B49" |
|  |  | **Bacteremia** | "018","0312","7907" | "A192","A198","A199","R7881" |
|  |  | **CNS infection** | "013","036","094","320","322","325","3240","3241","3249" | "A170","A171","A1781","A1782","A1789","A179","A390","A3981","A5211","A5213","A5214","A5219","A523","G000","G001","G002","G003","G01","G008","G009","G042","G030","G038","G031","G039","G08","G060","G061","G062" |
|  |  | **Soft tissue infection** | "015”,”017”,”0311”,”035","451”,”110”,”111”,”1123","681","682","683","686","7110","730" | "A1801”,”A1802”,”A1803”,”A5431”,”A5432”,”A5439”,”A5433”,”A5449”,”A5441”,”A5440”,”A545”,”A46","I8000”,”I8010”,”I80209”,”I803”,”I80219”,”I808”,”I809”,”B350”,”B351”,”B352”,”B356”,”B353”,”B354”,”B355","B358","B359","B360","B361","B362","B363","B368","B369","B372","L03019","L03029","L03039","L03049","K122","L03211","L03212","L03213","L03221","L03222","L03319","L03329","L03129","L03119","L03317","L03811”,”L03818”,”L03891”,”L03898”,”L0390”,”L0391”,”L049”,”L080”,”L88”,”L0889”,”L980”,”L089","M0000”,”M0010”,”M0020”,”M0080”,”M009”,”M00019”,”M00219”,”M00819”,”M00029”,”M00129","M00229","M00829","M00039","M00139","M00239","M00839","M00049","M00149","M00249","M00849","M00059","M00159","M00259","M00859","M00069","M00169","M00269","M00869","M00079","M00179","M00279","M00879","M0008","M0018","M0028","M0088","M0009","M0019","M0029","M0089","M8610","M8620","M86119","M86219","M86129","M86229","M86139","M86239","M86149,M86249,M86159","M86259","M86169","M86269","M86179","M86279","M8618","M8628","M8619","M8629","M8660","M86619","M86629","M86639","M86642","M86659","M86669","M86679","M8668","M8669","M869","M4620","M8960","M89619","M89629","M89639","M89649","M89659","M89669","M89679","M8968","M8969","M9080","M90819","M90829","M90839","M90849","M90859","M90869","M90879","M9088","M9089","M4630" |
|  |  | **Upper respiratory infection** | "461","462","463","464","465" | "J0100","J0110”,”J0120”,”J0130”,”J0140”,”J0190”,”J029”,”J0390”,”J040”,”J050”,”J0410”,”J0411”,”J042","J0510”,”J0511”,”J0430”,”J0431”,”J060”,”J069”,”A000”,”A001”,”A009”,”A0100”,”A011","A012”,”A013”,”A014”,”A030”,”A031”,”A032”,”A033”,”A038”,”A039”,”A050”,”A051”,”A052”,”A058”,”A053","A055”,”A054”,”A059”,”A044”,”A040”,”A041”,”A042”,”A043”,”A048”,”A045”,”A046”,”A049”,”A080”,”A082","A0811”,”A0819”,”A0831”,”A0832”,”A0839”,”A088”,”A09”,”A200”,”A201”,”A207”,”A202”,”A208”,”A209","A210”,”A213”,”A212”,”A211”,”A217”,”A218”,”A219”,”A220”,”A221”,”A222”,”A227”,”A228”,”A229”,”A230","A231”,”A232”,”A233”,”A238”,”A239”,”A240”,”A243”,”A249”,”A250”,”A251”,”A259”,”A3211”,”A3212”,”A327","A3281”,”A3289”,”A329”,”A267”,”A268”,”A269”,”A280”,”A288”,”A289”,”A360”,”A361”,”A3689”,”A362","A3686”,”A3681”,”A3685”,”A363”,”A3682”,”A3683”,”A3684”,”A369”,”A3700”,”A3710”,”A3780”,”A3790","L081”,”A420”,”A421”,”A422”,”B479”,”A4281”,”A4282”,”A4289”,”A438”,”A429”,”A439”,”B471”,”A480","A488”,”K9081”,”A4851”,”A4852”,”M60009”,”A483”,”B955”,”B950”,”B951”,”B954”,”B952”,”B958”,”B9561","B9562”,”B957”,”B953”,”B961”,”B9621”,”B9622”,”B9623”,”B9620”,”B9629”,”B963”,”B964”,”B965”,”A493","B960”,”B966”,”B967”,”B9689”,”B9681”,”A5009”,”A501”,”A502”,”A5031”,”A5040”,”A5045”,”A5042","A5041”,”A5049”,”A5052”,”A5057”,”A5059”,”A506”,”A507”,”A509”,”A510”,”A511”,”A512”,”A5131”,”A5139","A5149”,”A5143”,”A5146”,”A5145”,”A5141”,”A5132”,”A515”,”A5201”,”A5202”,”A5203”,”A5206”,”A5209","A5200”,”A5271”,”A5272”,”A5274”,”A5275”,”A5277”,”A5278”,”A5273”,”A5276”,”A5279”,”A528”,”A529","A530”,”A539”,”A270”,”A2781”,”A2789”,”A279”,”A690”,”A691”,”A660”,”A661”,”A662”,”A663”,”A664”,”A665","A666”,”A667”,”A668”,”A669”,”A670”,”A671”,”A672”,”A673”,”A679”,”A65”,”A698”,”A699”,”J441”,”J479","J471”,”J860”,”J869”,”J850”,”J851”,”J852”,”J853”,”T8579XA”,”T826XXA”,”T827XXA”,”T85730A","T85731A”,”T85732A”,”T85733A”,”T85734A”,”T85735A”,”T85738A”,”T83510A”,”T83511A”,”T83512A","T83518A”,”T83590A”,”T83591A”,”T83592A”,”T83593A”,”T83598A”,”T8361XA”,”T8362XA”,”T8369XA","T8450XA”,”T8460XA”,”T847XXA”,”T8571XA”,”T814XXA”,”K6811”,”T80219A”,”T80211A”,”T80212A","T8022XA”,”T8029XA”,”T880XXA" |

**Table S3: Missing rate for demographics and clinical variables extracted from the database during the observation period.**

| Variable | Number of missing | Percent of missing (%) |
| --- | --- | --- |
| NLR | 9815 | 78.89 |
| Serum albumin concentration | 7525 | 60.49 |
| PaO_2_/FiO_2_ ratio | 7176 | 57.68 |
| Serum lactate concentration | 7120 | 57.23 |
| Base excess | 5670 | 45.58 |
| PaO_2_ | 5670 | 45.58 |
| PaCO_2_ | 5670 | 45.58 |
| pH | 5670 | 45.58 |
| ALT | 3118 | 25.06 |
| AST | 3073 | 24.70 |
| Total bilirubin concentration | 3024 | 24.31 |
| aPTT | 2081 | 16.73 |
| INR | 1801 | 14.48 |
| Fluid balance | 1438 | 11.56 |
| Urine output | 1370 | 11.01 |
| RDW | 520 | 4.18 |
| Hemoglobin concentration | 477 | 3.83 |
| Platelet | 473 | 3.80 |
| WBC | 465 | 3.74 |
| Glucose | 452 | 3.63 |
| HCT | 440 | 3.54 |
| Bicarbonate | 435 | 3.50 |
| Creatinine | 434 | 3.49 |
| BUN | 429 | 3.45 |
| Temperature | 426 | 3.42 |
| Chloride | 421 | 3.38 |
| Sodium | 418 | 3.36 |
| SAP | 341 | 2.74 |
| DAP | 341 | 2.74 |
| GCS | 328 | 2.64 |
| SOFA score | 163 | 1.31 |
| SpO_2_ | 127 | 1.02 |
| MAP | 125 | 1.00 |
| RR | 115 | 0.92 |
| HR | 96 | 0.77 |

SOFA = Sequential Organ Failure Assessment; WBC = White blood cells; RDW = Red blood cell distribution width; NLR = Neutrophil to lymphocyte ratio; HCT = Hematocrit; ALT = Alanine transaminase; AST = aspartate aminotransferase; GCS = Glasgow Coma Scale; INR = International normalized ratio; aPTT = activated Partial Thromboplastin Clotting Time; BUN = Blood Urea Nitrogen; SAP = Systolic Arterial Pressure; MAP = Mean Arterial Pressure; DAP = Diastolic Arterial Pressure; SpO_2 =_ Peripheral capillary oxygen saturation; PaO_2_ = Partial Pressure of Oxygen; PaCO_2_ = Partial Pressure of Carbon Dioxide; PaO_2_/FiO_2_ ratio= Ratio of arterial oxygen partial pressure to fractional inspired oxygen; RR = Respiration rate; HR = Heart rate.

**Table S4: Missing rate for demographics and clinical variables extracted from the database on the first day.**

| Variable | Number of missing | Percent of missing (%) |
| --- | --- | --- |
| NLR | 1289 | 56.91 |
| PaO_2_/FiO_2_ ratio | 1182 | 52.19 |
| Height | 970 | 42.83 |
| Serum albumin concentration | 882 | 38.94 |
| Serum lactate concentration | 802 | 35.41 |
| Base excess | 676 | 29.85 |
| PaO_2_ | 676 | 29.85 |
| PaCO_2_ | 676 | 29.85 |
| pH | 676 | 29.85 |
| ALT | 297 | 13.11 |
| AST | 291 | 12.85 |
| Total bilirubin concentration | 290 | 12.80 |
| aPTT | 127 | 5.61 |
| INR | 96 | 4.24 |
| Urine output | 94 | 4.15 |
| Fluid balance | 83 | 3.66 |
| Weight | 68 | 3.00 |
| Temperature | 28 | 1.24 |
| RDW | 23 | 1.02 |
| Platelet | 21 | 0.93 |
| Hemoglobin | 20 | 0.88 |
| WBC | 18 | 0.79 |
| HCT | 16 | 0.71 |
| Glucose | 10 | 0.44 |
| SAP | 9 | 0.40 |
| DAP | 9 | 0.40 |
| Bicarbonate | 8 | 0.35 |
| Chloride | 7 | 0.31 |
| Sodium | 7 | 0.31 |
| Creatinine | 7 | 0.31 |
| BUN | 7 | 0.31 |
| RR | 2 | 0.09 |

SOFA = Sequential Organ Failure Assessment; WBC = White blood cells; RDW = Red blood cell distribution width; NLR = Neutrophil to lymphocyte ratio; HCT = Hematocrit; ALT = Alanine transaminase; AST = aspartate aminotransferase; GCS = Glasgow Coma Scale; INR = International normalized ratio; aPTT = activated Partial Thromboplastin Clotting Time; BUN = Blood Urea Nitrogen; SAP = Systolic Arterial Pressure; MAP = Mean Arterial Pressure; DAP = Diastolic Arterial Pressure; SpO_2 =_ Peripheral capillary oxygen saturation; PaO_2_ = Partial Pressure of Oxygen; PaCO_2_ = Partial Pressure of Carbon Dioxide; PaO_2_/FiO_2_ ratio = Ratio of arterial oxygen partial pressure to fractional inspired oxygen; RR = Respiration rate; HR = Heart rate.

**Table S5: Thresholds for clinical variables as albumin therapy indication.**

| Variable | Threshold |
| --- | --- |
| serum albumin concentration | < 2.5 g/dL |
|  | < 3.0 and ≥ 2.5 g/dL |
|  | < 3.5 and ≥ 3.0 g/dL |
|  | < 4.0 and ≥ 3.5 g/dL |
|  | ≥ 4.0 g/dL |
| serum lactate concentration | < 2.0 mmol/L |
|  | < 3.0 and ≥ 2.0 mmol/L |
|  | < 4.0 and ≥ 3.0 mmol/L |
|  | ≥ 4.0 mmol/L |
| MAP | < 55 mmHg |
|  | < 60 and ≥ 55 mmHg |
|  | < 65 and ≥ 60 mmHg |
|  | < 70 and ≥ 65 mmHg |
|  | ≥ 70 mmHg |
| NEE equivalent dose | < 0.1 and > 0 mcg/kg/min |
|  | < 0.2 and ≥ 0.1 mcg/kg/min |
|  | < 0.3 and ≥ 0.2 mcg/kg/min |
|  | < 0.4 and ≥ 0.3 mcg/kg/min |
|  | ≥ 0.4 mcg/kg/min |

MAP = Mean Arterial Pressure; NEE = Norepinephrine Equivalence.

**Table S6: Multivariable Cox model with time-fixed and time-dependent covariates for 28-day mortality adding to the model the effect of albumin administration, weighted with marginal structural models.**

| Variable | HR | Lower limit of 95% CI | Upper limit of 95% CI | *P* value |
| --- | --- | --- | --- | --- |
| Albumin administration | 0.64 | 0.51 | 0.80 | < 0.001 |
| RDW | 1.06 | 1.02 | 1.10 | 0.003 |
| Total bilirubin | 1.04 | 1.03 | 1.05 | < 0.001 |
| GCS | 0.94 | 0.92 | 0.96 | < 0.001 |
| Platelet | 1.02 | 1.00 | 1.05 | 0.02 |
| aPTT | 1.04 | 1.00 | 1.07 | 0.04 |
| MAP | 0.98 | 0.97 | 0.98 | < 0.001 |
| Base excess | 0.91 | 0.88 | 0.94 | < 0.001 |
| Bicarbonate | 1.06 | 1.02 | 1.10 | < 0.001 |
| Serum lactate concentration | 1.15 | 1.11 | 1.19 | < 0.001 |
| PaO_2_ | 1.03 | 1.02 | 1.05 | < 0.001 |
| Fluid balance | 0.97 | 0.95 | 1.00 | 0.05 |
| Respiratory SOFA | 0.91 | 0.84 | 0.99 | 0.02 |
| Gender | 1.33 | 1.10 | 1.61 | 0.003 |
| Admission age | 1.03 | 1.02 | 1.04 | < 0.001 |

Covariates: time-fixed: admission age, gender; time-varying: RDW, total bilirubin, GCS score, platelet (20*10^9/L increase), aPTT (10 s increase), MAP, base excess, bicarbonate, serum lactate concentration, PaO_2_ (10 mmHg increase), fluid balance (1000 ml increase), respiratory SOFA.

**Table S7: Association between albumin administration and 28-day mortality estimated by extended Cox regression model with time-varying covariates.**

| Variables | Hazard ratio | Lower limit of 95% CI | Upper limit of 95% CI | *P* value |
| --- | --- | --- | --- | --- |
| Albumin administration | 0.74 | 0.61 | 0.89 | 0.001 |
| Age (with every 10-year increase) | 1.16 | 1.09 | 1.24 | < 0.001 |
| Gender (female as reference) | 1.31 | 1.12 | 1.53 | < 0.001 |
| **Admission type (elective as reference)** | | | | |
| Emergency | 2.90 | 0.74 | 11.45 | 0.13 |
| Observation | 2.95 | 0.73 | 11.85 | 0.13 |
| Surgical | 0.51 | 0.08 | 3.33 | 0.48 |
| Urgent | 2.93 | 0.74 | 11.63 | 0.13 |
| Serum albumin concentration at presentation | 0.88 | 0.79 | 0.98 | 0.016 |
| **Ethnicity (Asian as reference)** | | | | |
| Black | 0.67 | 0.37 | 1.21 | 0.18 |
| Hispanic | 1.04 | 0.57 | 1.89 | 0.91 |
| White | 0.88 | 0.52 | 1.50 | 0.64 |
| Others | 0.90 | 0.52 | 1.56 | 0.71 |
| **Infection site (respiratory as reference)** | | | | |
| Gastrointestinal | 0.82 | 0.59 | 1.13 | 0.22 |
| Genito-urinary | 1.00 | 0.72 | 1.38 | 0.98 |
| Others | 1.09 | 0.91 | 1.32 | 0.36 |
| Serum lactate concentration | 1.15 | 1.13 | 1.18 | < 0.001 |
| SOFA score | 1.00 | 0.98 | 1.02 | 0.77 |
| PaO_2_/FiO_2_ ratio (with every 50-mmHg increase) | 1.07 | 1.03 | 1.10 | < 0.001 |
| Vasopressor dose | 1.01 | 1.00 | 1.02 | 0.14 |
| Urine output (with every 1-L increase) | 0.49 | 0.42 | 0.58 | < 0.001 |
| MAP | 0.98 | 0.97 | 0.98 | < 0.001 |

CI = Confidence interval; SOFA = Sequential Organ Failure Assessment; MAP = Mean Arterial Pressure.

Note: Time-fixed covariates were measured in the first 24 hours after ICU admission, and time-varying covariates were measured daily during the ICU stay.

**Table S8: Univariate and multivariate analyses weighted with MSCM of the association between albumin infusion and 28-day mortality in patients after the exclusion of spontaneous bacterial peritonitis.**

| Univariate | | | multivariate | | |
| --- | --- | --- | --- | --- | --- |
| Variable | HR (95% CI) | *P* value | Variable | HR (95% CI) | *P* value |
| Albumin infusion | 0.72 (0.56-0.92) | 0.009 | Albumin administration | 0.62 (0.49-0.80) | < 0.001 |
| - | - | - | RDW | 1.05 (1.01-1.10) | 0.013 |
| - | - | - | Total bilirubin | 1.04 (1.03-1.05) | < 0.001 |
| - | - | - | GCS | 0.94 (0.91-0.96) | < 0.001 |
| - | - | - | Platelet | 1.02 (1.00-1.04) | 0.11 |
| - | - | - | aPTT | 1.03 (0.99-1.07) | 0.11 |
| - | - | - | MAP | 0.98 (0.97-0.99) | < 0.001 |
| - | - | - | Base excess | 0.91 (0.88-0.94) | < 0.001 |
|  |  |  | Bicarbonate | 1.05 (1.01-1.09) | 0.007 |
|  |  |  | Serum lactate concentration | 1.15 (1.11-1.19) | < 0.001 |
|  |  |  | PaO_2_ | 1.03 (1.01-1.05) | 0.005 |
|  |  |  | Fluid balance | 0.97 (0.95-1.00) | 0.08 |
|  |  |  | Respiratory SOFA | 0.92 (0.84-1.00) | 0.06 |
|  |  |  | Gender | 1.30 (1.06-1.60) | 0.012 |
|  |  |  | Admission age | 1.03 (1.02-1.04) | < 0.001 |

Covariates: time-fixed: admission age, gender; time-varying: RDW, total bilirubin, GCS score, platelet (20*10^9/L increase), aPTT (10 s increase), MAP, base excess, bicarbonate, serum lactate concentration, PaO_2_ (10 mmHg increase), fluid balance (1000 ml increase), respiratory SOFA.

**Table S9: Multivariable Cox model with time-fixed and time-dependent covariates for 28-day mortality adding to the model the effect of albumin administration, weighted with marginal structural models.**

**The analysis was repeated until reaching the highest level of serum albumin concentration, for which the albumin infusion still showed a positive effect on the outcome.**

| Serum albumin concentration cut-off | Hazard ratio | Lower limit of 95% CI | Upper limit of 95% CI | *P* value |
| --- | --- | --- | --- | --- |
| 2.7 g/dL | 0.67 | 0.46 | 0.96 | 0.03 |
| 2.8 g/dL | 0.72 | 0.51 | 1.01 | 0.06 |

Note: Covariates: time-fixed: admission age, gender; time-varying: RDW, total bilirubin, GCS score, platelet (20*10^9/L increase), aPTT (10 s increase), MAP, base excess, bicarbonate, serum lactate concentration, PaO_2_ (10 mmHg increase), fluid balance (1000 ml increase), respiratory SOFA.

**Table S10: Multivariable Cox model with time-fixed and time-dependent covariates for 28-day mortality adding to the model the effect of albumin administration, weighted with marginal structural models.**

**The analysis was repeated until reaching the lowest level of serum lactate concentration, for which the albumin infusion still showed a positive effect on the outcome.**

| Serum lactate concentration cut-off | Hazard ratio | Lower limit of 95% CI | Upper limit of 95% CI | *P* value |
| --- | --- | --- | --- | --- |
| 2.2 mmol/L | 0.63 | 0.41 | 0.98 | 0.03 |
| 2.1 mmol/L | 0.70 | 0.45 | 1.11 | 0.13 |

Note: Covariates: time-fixed: admission age, gender; time-varying: RDW, total bilirubin, GCS score, platelet (20*10^9/L increase), aPTT (10 s increase), MAP, base excess, bicarbonate, serum lactate concentration, PaO_2_ (10 mmHg increase), fluid balance (1000 ml increase), respiratory SOFA.

**Table S11: Correlation between the types of albumin solutions administered and 28-day mortality as estimated by the MSCM.**

| Albumin solution type^*^ | No. of patients | Hazard ratio | Lower limit of 95% CI | Upper limit of 95% CI | *P* value |
| --- | --- | --- | --- | --- | --- |
| 25% | 918 | 0.72 | 0.53 | 0.97 | 0.031 |
| 5% | 442 | 0.38 | 0.22 | 0.65 | < 0.001 |

* In our cohort, we extracted a total of 8,308 albumin administrations, of which 1,617 (19%) were 5% albumin and 6,691 (81%) were 25% albumin.

CI = Confidence interval; MSCM = Marginal Structural Cox proportional hazards Model.

**Figure S1: Directed acyclic graph (DAG) illustrating the potential actions of confounding covariates on the relation between the administration of albumin according to different albumin concentrations and clinical outcomes.
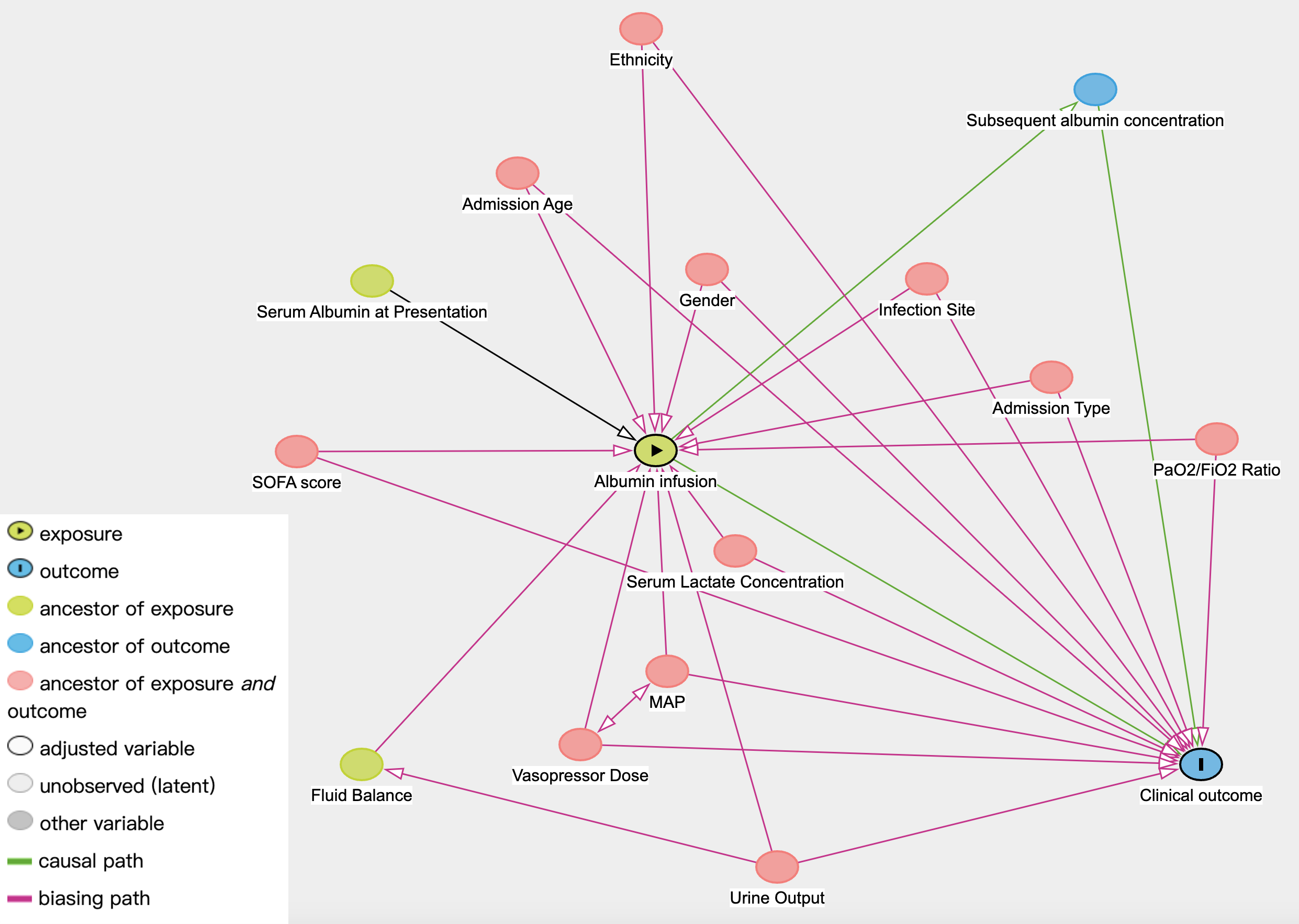
**

SOFA = Sequential Organ Failure Assessment; MAP = Mean Arterial Pressure.


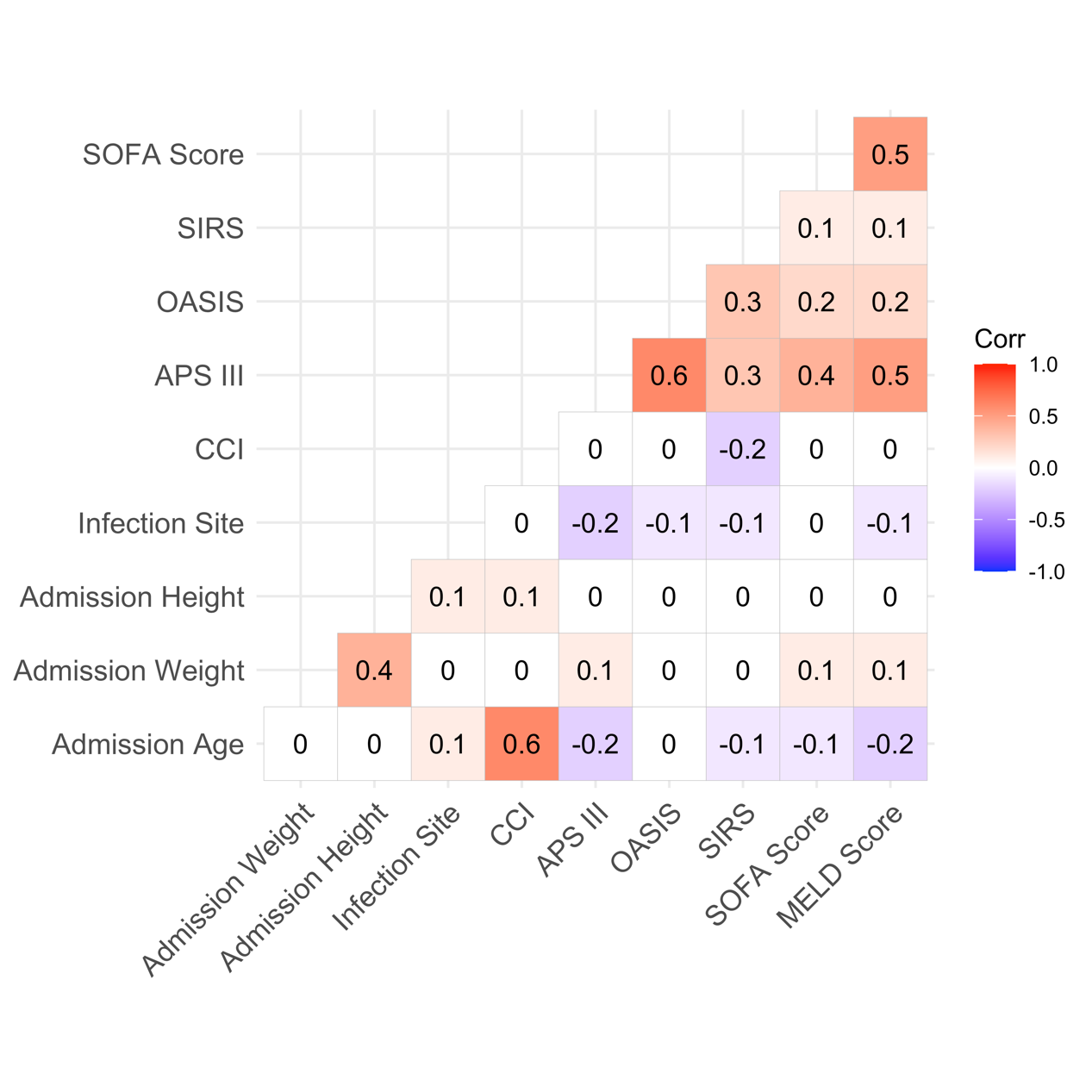
**Figure S2: Correlation matrix for time-fixed covariates.**

SOFA = Sequential Organ Failure Assessment; CCI = Charlson Comorbidity Index; APS III = Simplified Acute Physiology Score III; OASIS = Oxford Acute Severity of Illness Score; SIRS = Systemic inflammatory response syndrome; MELD = Model for End-stage Liver Disease.

**Figure S3: Correlation matrix for time-dependent covariates.**


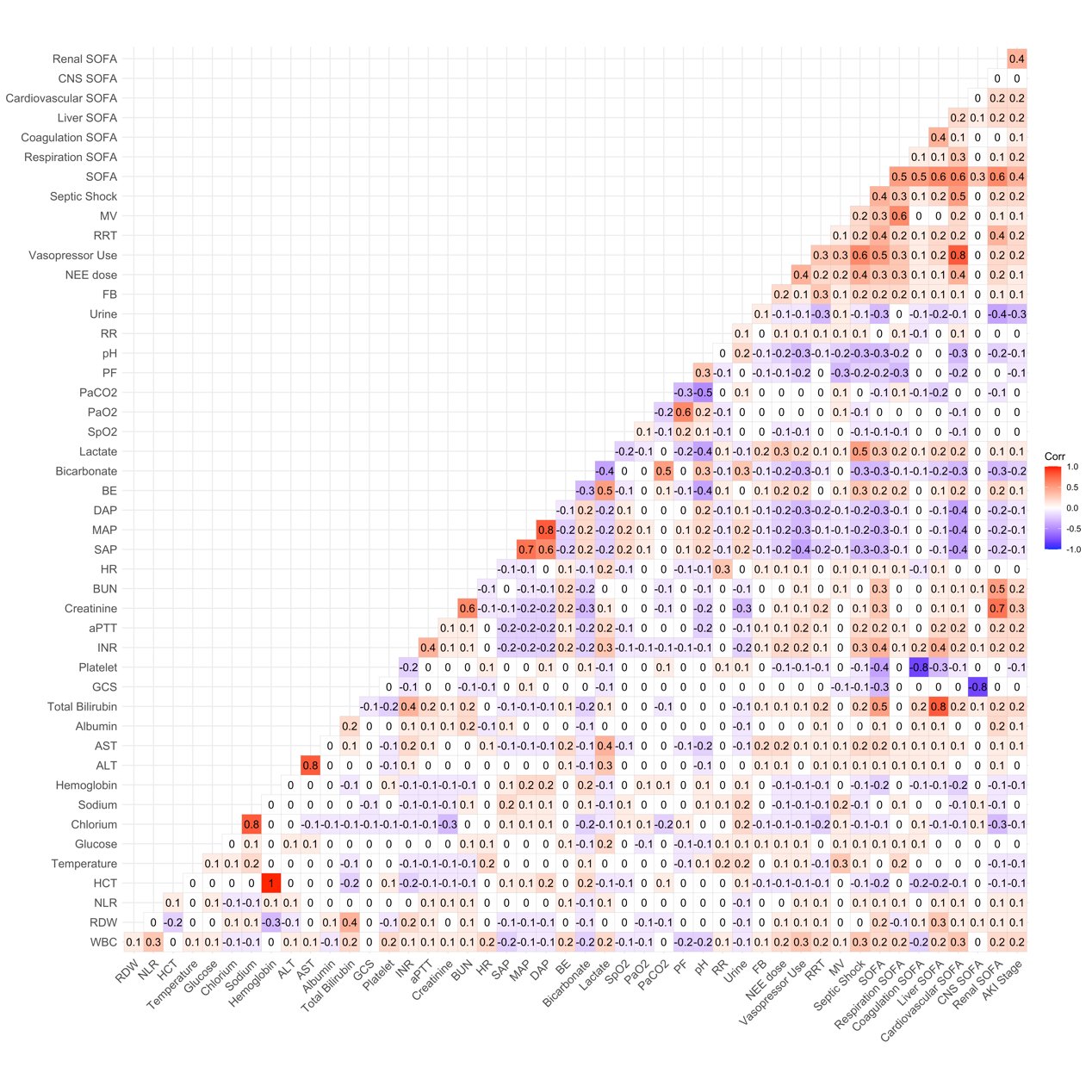


WBC = White blood cells; NLR = Neutrophil to lymphocyte ratio; ALT = Alanine transaminase; AST = aspartate aminotransferase; GCS = Glasgow Coma Scale; INR = International normalized ratio; aPTT = activated Partial Thromboplastin Clotting Time; BUN = Blood Urea Nitrogen; MAP = Mean Arterial Pressure; FB = Fluid balance; BE = Base excess; PaCO_2_ = Partial Pressure of Carbon Dioxide; PaO_2_/FiO_2_ ratio = Ratio of arterial oxygen partial pressure to fractional inspired oxygen; RR = Respiration rate; RRT = Renal Replacement Therapy.

**Figure S4: Variable selection for Cox time-dependent and time-independent model factors associated with 28-day mortality.**

**
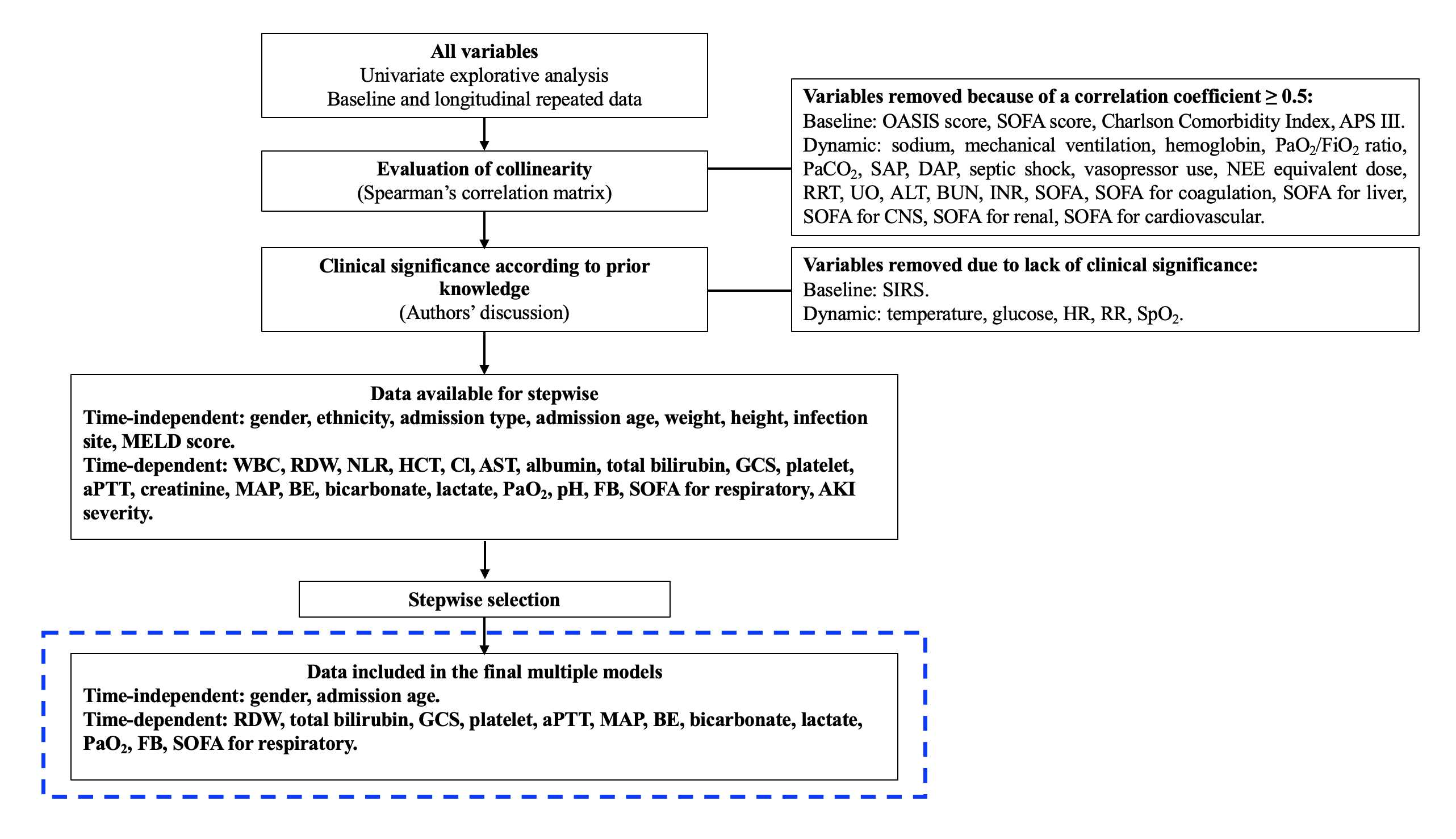
**

**Figure S5: Schematic illustrations of the sub-cohort establishment.**

**
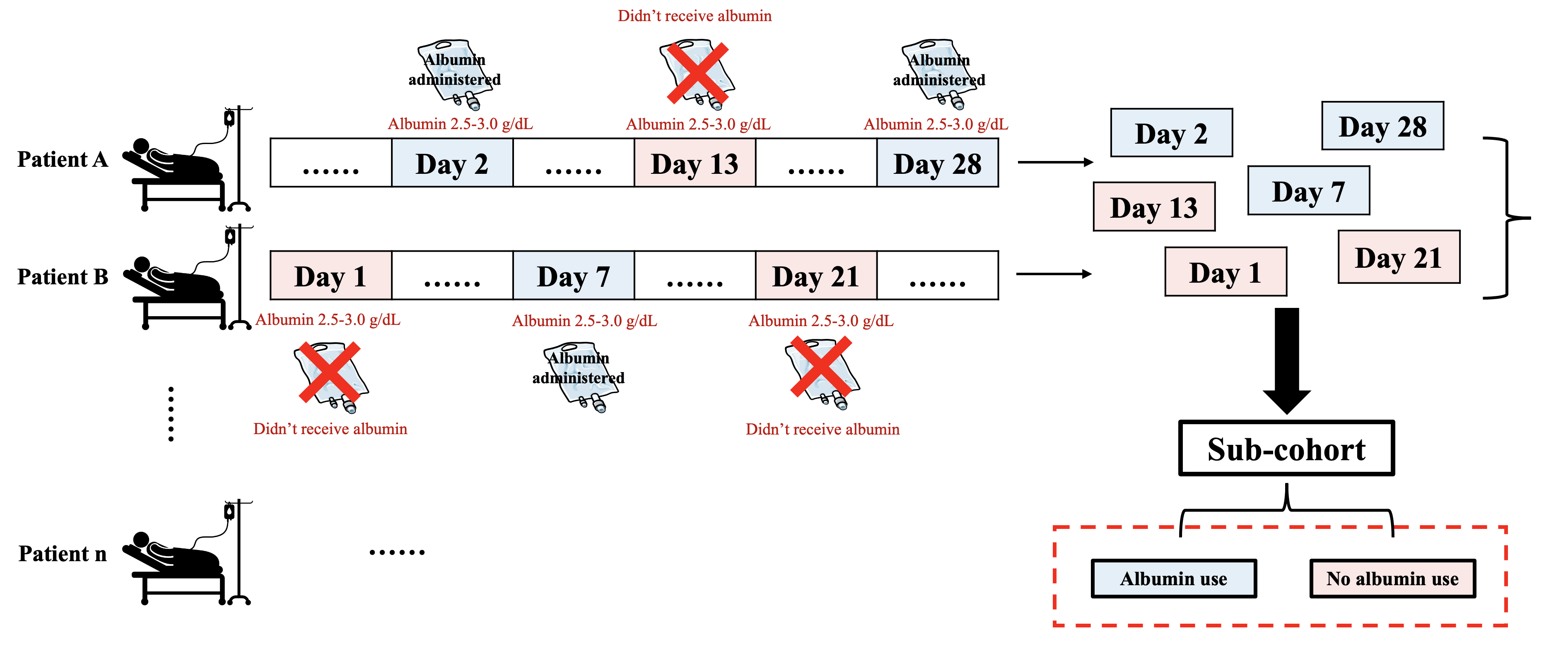
**

**Figure S6: Expanded Kaplan-Meier survival analysis, weighted by marginal structural models.**

**
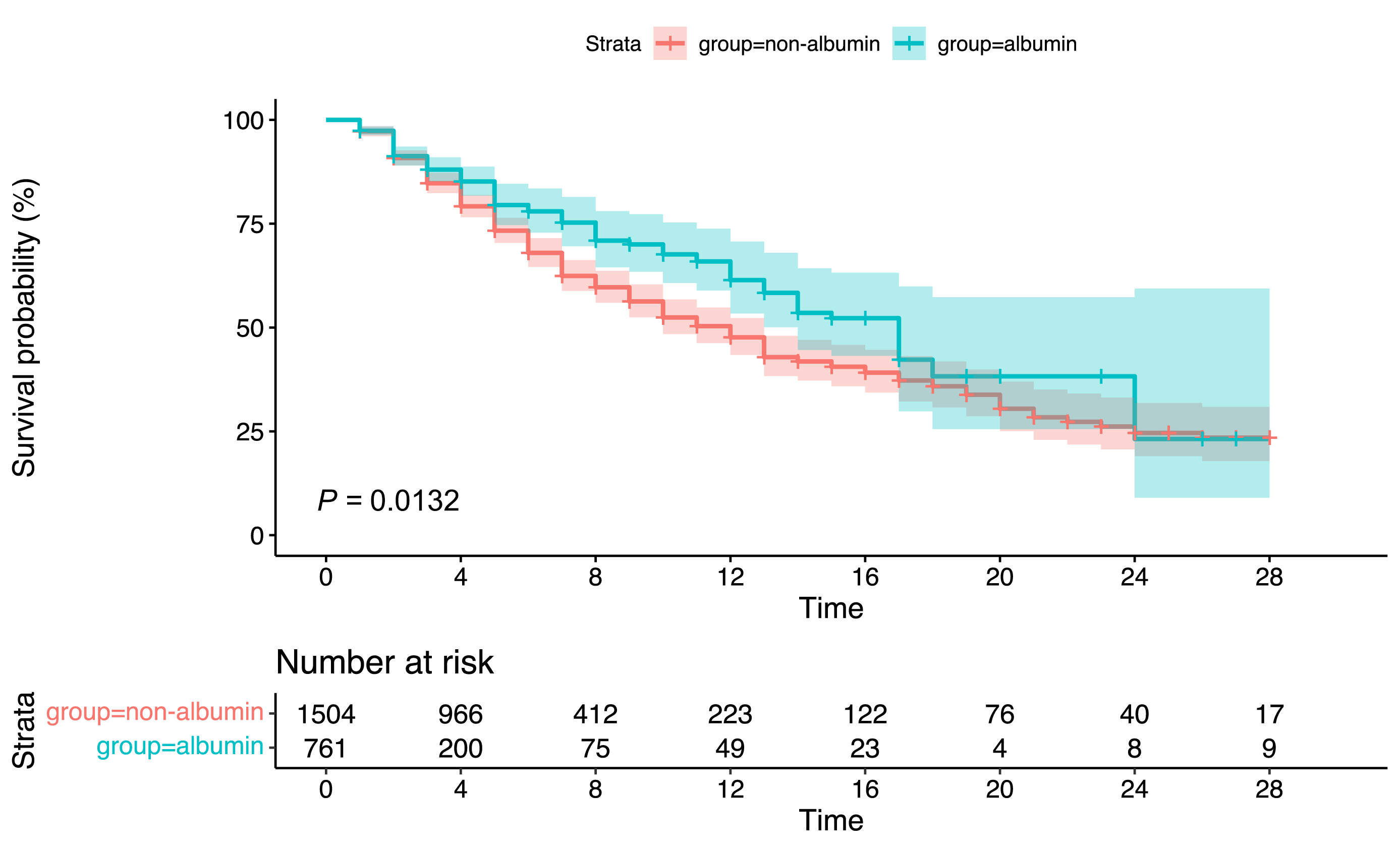
**


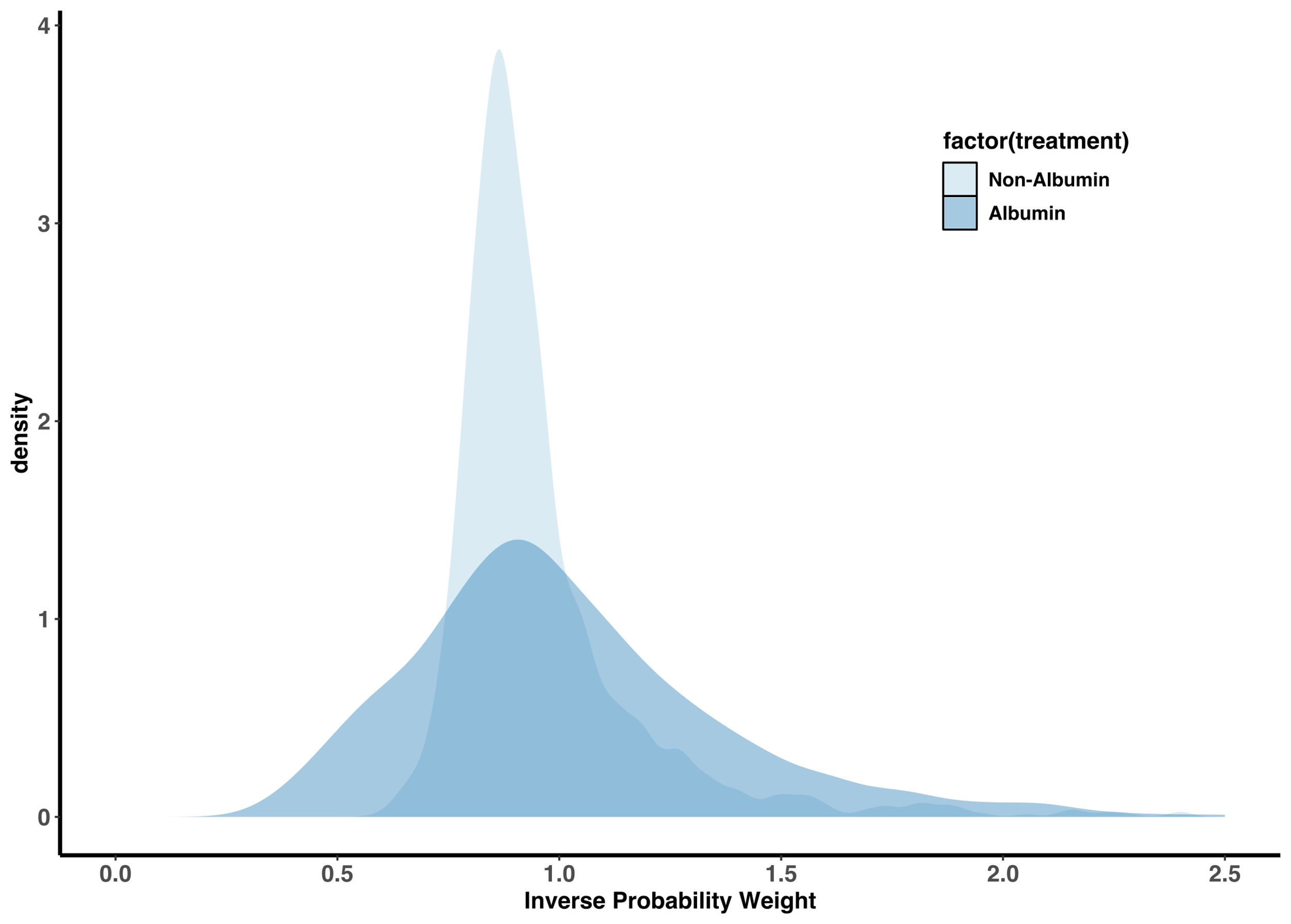
**Figure S7: Distribution of inverse probability weight in albumin and non-albumin groups.**


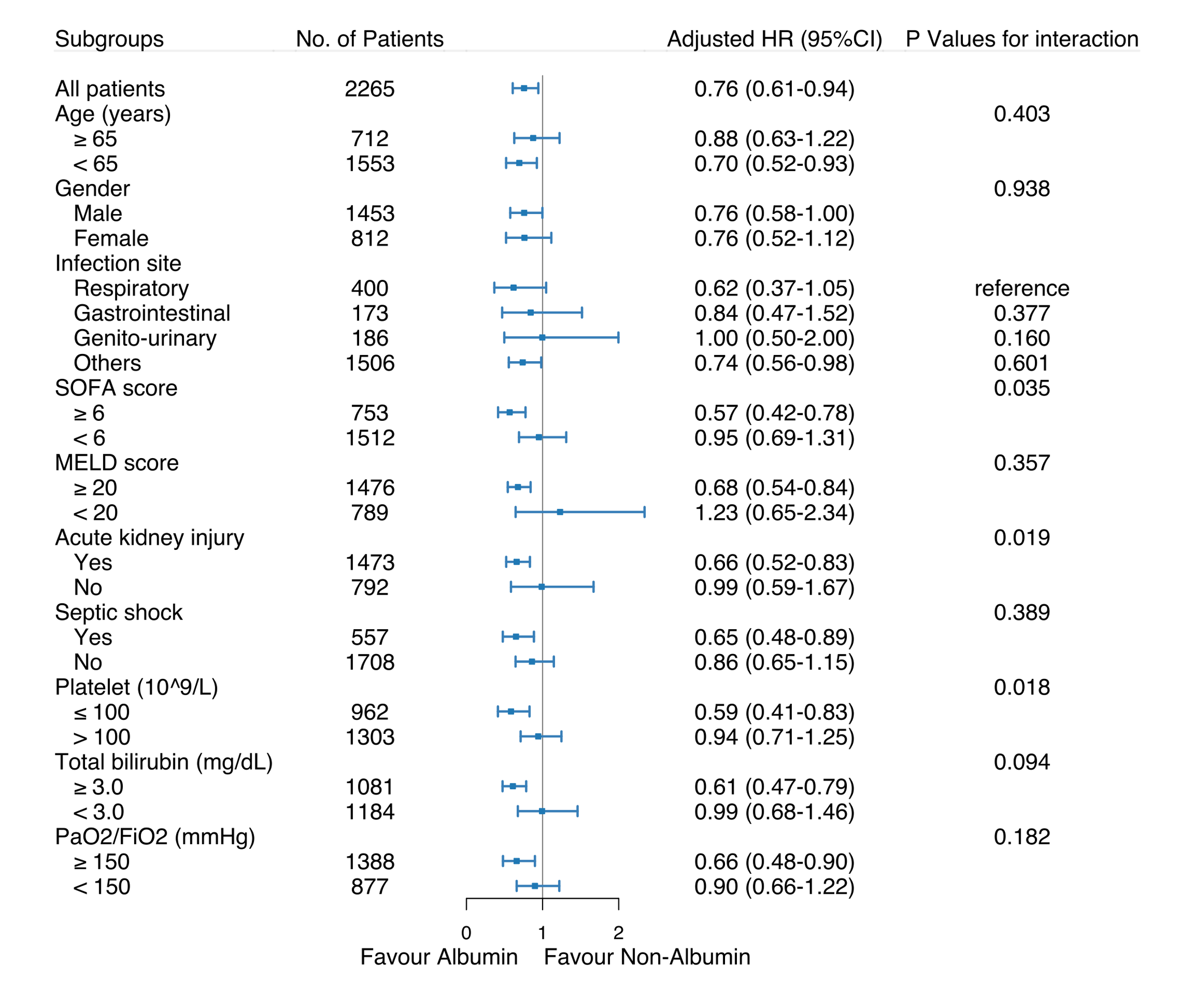
**Figure S8: Adjusted risk of death at 28 days measured by Marginal Structural Cox proportional hazards Model according to baseline subgroups.**

SOFA = Sequential Organ Failure Assessment; MELD = Model for End-stage Liver Disease; HR = Hazard ratio; CI = Confidence interval.
